# Molecular palaeontology illuminates the evolution of ecdysozoan vision

**DOI:** 10.1098/rspb.2018.2180

**Published:** 2018-12-05

**Authors:** James F. Fleming, Reinhardt Møbjerg Kristensen, Martin Vinther Sørensen, Tae-Yoon S. Park, Kazuharu Arakawa, Mark Blaxter, Lorena Rebecchi, Roberto Guidetti, Tom A. Williams, Nicholas W. Roberts, Jakob Vinther, Davide Pisani

**Affiliations:** 1 School of Earth Sciences, University of Bristol, Queen's Road, Bristol, UK; 2 Natural History Museum of Denmark, Copenhagen, Denmark; 3 Division of Polar Earth-System Sciences, Korea Polar Research Institute, Incheon 21990, Republic of Korea; 4 Institute for Advanced Biosciences, Keio University, Tsuruoka, Yamagata, Japan; 5 Institute of Evolutionary Biology, School of Biological Sciences, University of Edinburgh, Edinburgh, UK; 6 Department of Life Sciences, University of Modena and Reggio Emilia, Via G. Campi 213/D, Modena, Italy; 7 School of Biological Sciences, University of Bristol, Tyndall Avenue, Bristol, UK

**Keywords:** opsin, phylogeny, vision, evolution

## Abstract

Colour vision is known to have arisen only twice—once in Vertebrata and once within the Ecdysozoa, in Arthropoda. However, the evolutionary history of ecdysozoan vision is unclear. At the molecular level, visual pigments, composed of a chromophore and a protein belonging to the opsin family, have different spectral sensitivities and these mediate colour vision. At the morphological level, ecdysozoan vision is conveyed by eyes of variable levels of complexity; from the simple ocelli observed in the velvet worms (phylum Onychophora) to the marvellously complex eyes of insects, spiders, and crustaceans. Here, we explore the evolution of ecdysozoan vision at both the molecular and morphological level; combining analysis of a large-scale opsin dataset that includes previously unknown ecdysozoan opsins with morphological analyses of key Cambrian fossils with preserved eye structures. We found that while several non-arthropod ecdysozoan lineages have multiple opsins, arthropod multi-opsin vision evolved through a series of gene duplications that were fixed in a period of 35–71 million years (Ma) along the stem arthropod lineage. Our integrative study of the fossil and molecular record of vision indicates that fossils with more complex eyes were likely to have possessed a larger complement of opsin genes.

## 1. Introduction

### (a) Background

Invertebrates with diverse body plans and ecologies dominated the ecosystems of the Cambrian [1–4]. Species from the superphylum Ecdysozoa, including Priapulida (penis worms) and Arthropoda (e.g. the extant crustaceans, insects, myriapods, and chelicerates) were particularly important constituents of these earliest complex ecosystems. The ability to perceive light and detect visual cues is important for extant animals, allowing them to regulate their circadian rhythms, inform visually guided behaviours such as predation, and communicate through visual signals [5,6]. Given the eye complexity of some Cambrian fossil species, such as *Anomalocaris* and the trilobites [7], and given that some of these fossils had structural colours [1], it is likely that vision played a role in shaping the Cambrian radiation of animals. Hence, understanding the visual ecology of important fossil taxa is fundamental to a greater comprehension of key evolutionary events such as the Cambrian explosion [4,8].

The Cambrian explosion is so named as it was characterized by an expansion of animal body plans, displaying also a diversity of complex sensory systems. Many of the most striking examples of this diversity are found in the stem arthropod lineage, i.e. among the taxa that diverged before the last common ancestor of the living arthropods (Chelicerata, Myriapoda, and Pancrustacea) but after the separation of the arthropod lineage from its closest relatives—Tardigrada and Onychophora. The stem arthropod lineage included many of the apex predators of the early Palaeozoic, such as *Anomalocaris*, *Opabinia*, and *Pambdelurion* [9], and over this period, these taxa developed a number of innovations, expanding the gut for improved digestion [9,10], enlarging and experimenting with more efficient mouth parts [11–13], and increasing motility [14,15]. The stalked compound eye is an example of the complex sensory organs that can be seen relatively early in Cambrian stem arthropods, with more complex eye structures being found in fossils that are more closely related to the crown arthropods or that are nested within crown Arthropoda [16].

Colour vision is a key element of living arthropod predatory visual ecology. Arthropod colour vision is mediated by visual pigments that use rhabdomeric-type opsins (r-opsins) [17]. A single spectral sensitivity is not sufficient to see in colour, but possession of two or more visual pigments can allow the detection of colours by comparative processing [17,18]. While visual pigments do not fossilize, phylogenetic methods and molecular data have previously been used to infer the visual capabilities of long extinct taxa [19].

**Figure 1.**
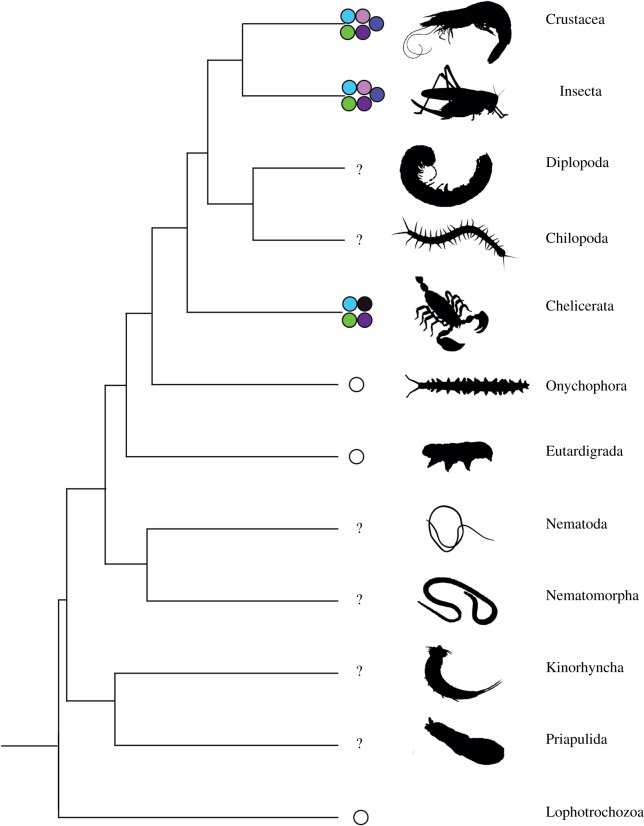
Distribution of well-characterized ecdysozoan opsins. Purple: short wave sensitive (SWS), violet: UV and Rh7, light blue: medium wave sensitive (MWS), and green: long wave sensitive (LWS). Black: UV/SWS. In all figures animal silhouettes are from www.phylopic.org.

The common ancestor of all living arthropods (i.e. the crown arthropod last common ancestor) minimally possessed four visual opsins (figure 1) [18]. These were the yellow-green (medium wave sensitive; MWS) opsin, the blue-green (long wave sensitive; LWS) opsin, Rhodopsin 7 (Rh7, which mediates circadian entrainment in *Drosophila* [20]), and the UV/SWS opsin, that in the opsin literature is generally referred to as the UV opsin (see §1b for guidelines on the gene nomenclature used in this paper). The UV/SWS gene underwent a duplication in the common ancestor of the allotriocarid pancrustaceans (insects, branchiopods, remipedes, and possibly cephalocarids) and of the malacostracans (e.g. lobsters and crabs) [18,21]. This duplication gave rise to an ultraviolet sensitive (UV) and a blue (short wave sensitive; SWS) opsin (figure 1) that are retained in the malacostracans [18,22,23] and allotriocarid lineages with a functional visual system (i.e. insects and branchiopods) [18,22,23]. Because the relationships between crustaceans are still partially unresolved [21,24–26], it is unclear where in the crustacean phylogeny there was a duplication of the UV/SWS gene. However, as Branchiopoda and Hexapoda are crown arthropods belonging to the Pancrustacea that diverged 497 Ma or more [24,25,27,28], we can be confident that this duplication happened in the Cambrian, within crown Arthropoda, after the separation of Myriapoda (e.g. centipedes and millipedes) from Pancrustacea [21].

While the duplication of the UV/SWS gene occurred within crown Arthropoda [22,23], the UV/SWS, MWS, LWS, and Rh7 paralogues evolved through a series of duplications that predate the origin of this group. Previous studies showed that while other ecdysozoans have r-opsins, they do not seem to have this set of paralogues [17,22,23].

We used newly sequenced transcriptomes and genomes, and publicly available data to identify a total of 218 ecdysozoan opsins to further investigate whether the ecdysozoan r-opsin diversification was an arthropod-specific phenomenon. We used dated phylogenies to investigate whether the evolution of the morphological and molecular components of ecdysozoan visual systems were, to some level, coupled. We compared the emergence of new opsin paralogues and the emergence of novel features in ecdysozoan eyes using key fossils and extant taxa. We included new data for the 518 Ma fossil *Pambdelurion whittingtoni*, which represents one of the oldest known occurrences of eye structures in the fossil record. We conclude that fossil ecdysozoans displaying greater eye complexity inherited more opsin paralogues, suggesting that the evolution of the molecular and morphological components of the ecdysozoan visual system may have been coupled.

### (b) Naming opsins

Describing the evolutionary history of a gene family is difficult when, as in the case of the opsin family, no clear nomenclatural schema has been applied consistently to newly identified genes. We have used a schema based on the regulatory gene literature [29,30] to name ancestral and paralogue genes (see electronic supplementary material and figure S1).

## 2. Material and methods

### (a) Identifying new r-opsins and assembly of an ecdysozoan visual opsins dataset

Raw transcriptome data publicly available on the National Centre for Biotechnology Information, Sequence Read Archive (NCBI SRA) archive for *Paragordius varius*, *Tubiluchus* sp*.*, *Ramazzottius varieornatus*, *Abacion magnum*, *Brachycybe lecontii*, *Cambala annulata*, *Cleidogona* sp*.*, *Craterostigmus tasmanianus*, *Eupolybothrus cavernicolus*, *Scutigera coleoptrata*, *Petaserpes* sp*.*, *Prostemmiulus* sp*.*, *Baetis* sp*.*, *Boreus hyemalis*, *Corydalus cornutus*, *Empusa pennata*, *Fopius arisanus*, *Haploembia palaui*, *Liposcelis entomophila*, *Meinertellus cundinamarcensis*, *Menopon gallinae*, *Periplaneta americana*, *Stylops melittae*, *Centruroides* sp*.*, *Frontinella* sp*.*, *Liphistius* sp., and *Neoscona arabesca* were assembled following the protocol presented in the electronic supplementary material. New data were generated for *Meiopriapulus fijiensis*, *Batillipes* sp., *Echiniscus testudo*, *Paramacrobiotus richtersi*, *Damon* sp., *Galeodes* sp*.*, *Limulus polyphemus*, *Neobisium carcinoides*, *Nymphon gracile*, *Oligolophus* sp., and *Oniscus* sp*.* following the protocols in the electronic supplementary material (see electronic supplementary material, table S1 for a list of all transcriptomes and genomes sequenced and their accession numbers).

Putative opsin sequences were identified from the assembled genomes and transcriptomes using a BLAST-based [31] approach (BLAST cut-off *E*-value = 10e−7). A previously collated set of 401 opsins from 117 species [23] was used as queries for the BLAST searches. To confirm that sequences identified were r-opsins, rather than members of other opsin families or more distantly related G-protein coupled receptors, the putative opsins were compared (using BLAST) to the NCBI non-redundant protein database. All sequences with a best match to an r-opsin were added to an existing dataset of 183 opsins (from [32,33]) made up of 179 well-characterized r-opsins (16 lophotrochozoan visual r-opsins, 7 vertebrate melanopsins, 16 arthropsins, 140 panarthropod visual r-opsins) and four cnidarian opsins. The cnidarian opsins are either sister to the other r-opsins [32,33] or they represent more distantly related, r-opsin-like genes [34] thus representing a valid outgroup to root our r-opsin phylogeny. The known and putative opsins were aligned in both MUSCLE [35] and PRANK [36]. Two initial opsin phylogenies (electronic supplementary material, figures S2 and S3) were derived in Phylobayes MPI 1.7 [37] under the GTR+G model [33] and rooted using the cnidarian opsins. The trees derived from the MUSCLE and PRANK alignments were not significantly different, and we focus on the MUSCLE alignment analyses below. Opsins that were more closely related to the panarthropod visual r-opsins than to any of the other opsins in the preliminary opsin tree, including new sequences from Priapulida and Nematomorpha, were confirmed to be members of the panarthropod visual r-opsin group and were retained for further analyses (electronic supplementary material, figures S2 and S3 and table S2). All alignments used in our study, and all new sequences, are available [38].

### (b) Ecdysozoan visual opsin phylogeny

A new alignment of 181 sequences was generated, including all the sequences that we identified to be more closely related to the arthropod visual r-opsins than to any other r-opsin, and a large sample of previously characterized panarthropod visual opsins [23]. This dataset was aligned using MUSCLE [35], and phylogenetic analyses were performed using a novel, r-opsin-specific GTR matrix derived following Abascal *et al*. [39], in PAML4.8 [40], which was implemented in Phylobayes MPI 1.7 [37]. The r-opsin-specific matrix is available [38]. In all phylogenetic analyses, site-specific rate heterogeneity was modelled using a Gamma distribution with four rate categories. Convergence was assessed by comparing the maximum discrepancies observed over the bipartitions and effective sample size using *Bpcomp* and *Tracecomp* [37]. For all analyses two independent chains were run. A burn-in of 50% of the sample size was used for all analyses, sampling every fiftieth generation following the burn-in period. Trees were rooted using the newly identified priapulid and nematomorph sequences (based on the relationships inferred for these sequences in phylogenetic analyses that included also more distantly related r-opsins).

Gene duplication events were inferred using ALE [41]. The positioning of these gene duplications along a rooted ecdysozoan phylogeny were compared with those proposed previously [22,23].

### (c) Estimating the time of fixation of opsin duplications

Divergence times between opsin genes were inferred using a relaxed, autocorrelated, molecular clock model (the Cox, Ingersoll, and Ross (CIR) process [42]) in Phylobayes v3.3 [37], with the root placed on the node separating the priapulid and nematomorph r-opsins from all other ecdysozoan r-opsins. Our new r-opsin GTR matrix was used to model the substitution process, with site-specific rate heterogeneity modelled using a Gamma distribution (four rate categories). The root prior was modelled using a Gamma distribution of average 600 Ma and standard deviation 26 Ma. These parameters generate a prior distribution for the root age of our r-opsin phylogeny congruent with the calibration interval for the origin of Ecdysozoa [24,43,44]. This prior distribution encompasses the currently accepted fossil maximum for the origin of Ecdysozoa (636.1 Ma; the maximum age of the Lantian biota) [24,43–45], and the accepted fossil minimum (541 Ma; the oldest traces of a priapulid-grade animal, *Treptichnus pedum* [46]). Fourteen node calibrations were applied to the tree (see electronic supplementary material, table S3), and the joint marginal priors were visualized before the analyses. Convergence was assessed using *Tracecomp* in Phylobayes.

### (d) Ecdysozoan divergence time analysis

An ecdysozoan timetree was generated that included both extant and fossil taxa using the total evidence tip dating method [47] under the fossilized birth death process [48] in RevBayes [49,50]. A molecular-morphological dataset was used for our analysis, combining the morphological data from [51], with six genes (2201 amino acid positions) from eight out of the 11 extant taxa in the dataset (all those for which some molecular data is available). The molecular data was either obtained from NCBI or from the transcriptomes we sequenced (see electronic supplementary material for accession numbers and [38]) for the data. The tree topology was fixed to reflect the phylogenetic tree of [51], but with the few polytomies in that tree resolved according to [52]. Fifty-one calibrations representing the age of the tip of every fossil species in the dataset (electronic supplementary material, table S4) were used. For the root prior, we used a soft-bounded uniform distribution with a lower bound of 542 Ma and an upper bound encompassing the age of the Lantian biota (636.1 Ma) as part of the 2.5% probability distribution allocated outside of the minimum–maximum boundary [24,43]. Absence of ecdysozoan fossils from the Lantian biota is considered to represent a true absence rather than lack of evidence for their presence [44,45]. The sampling prior for the extant taxa (*ρ*) was set to 0.00001, the ratio between the taxa in our dataset and the estimated diversity of the extant arthropods (approx. 10 000 000 species [53]). The exponential prior on the rate of fossil sampling (*ψ*) was set to 10, permitting very broad sampling of possible rates. The RevBayes Script used to run the analysis is available [38].

### (e) Divergence time comparison

Phylogenetic history and divergence times for the clades in our ecdysozoan tree were compared to the history of the duplications within the opsin family. Presence of an opsin in the taxa descending from an internal node in our ecdysozoan phylogeny was inferred by phylogenetic bracketing when all the descendants were extant taxa. If one or more of the descendants were fossils the presence of an opsin in the descendants was estimated by comparing the age of the considered clade in the ecdysozoan timetree against the age of the opsin duplication. A fossil is always younger than the clade it belongs to. Accordingly, fossil ages underestimate clade ages [54], by comparing two timetrees rather than the age of gene duplications against the fossil record we avoided making invalid comparisons. In cases where the lower bound of the 95% CI of the species divergence time occurred after and did not overlap with the upper bound of the 95% CI of the opsin duplication time, the duplication was inferred to have predated the divergence of the taxa descending from that node (with *p* = 0.95). The descending lineages were thus inferred to have inherited the two paralogues that emerged from the duplication. When the credibility intervals of a given speciation and duplication event overlapped, the species divergence might have predated the opsin duplication. In these cases we conservatively assumed that the lineages descending from the speciation event inherited the ancestral opsin gene rather than the paralogues that emerged from the duplication. Accordingly, our estimates for the number of paralogues that could have been inherited by fossil taxa are always minimal.

## 3. Results and discussion

### (a) Identifying new ecdysozoan opsins

We identified a total of 56 new r-opsins across Ecdysozoa (see electronic supplementary material, tables S1 and S2). While most new r-opsins were found within Arthropoda, 16 were found in other phyla. We identified two and four r-opsins in the priapulid *M. fijiensis* and in the nematomorph *P. varius*, respectively. In the tardigrades we identified multiple r-opsins in two heterotardigrade species and one r-opsin in a eutardigrade. In arthropods, new r-opsins were identified in six myriapod species (6 r-opsins), five chelicerates (6 r-opsins), and 12 pancrustaceans (28 r-opsins), see electronic supplementary material, table S2 for details.

### (b) Understanding the new ecdysozoan opsins

**Figure 2.**
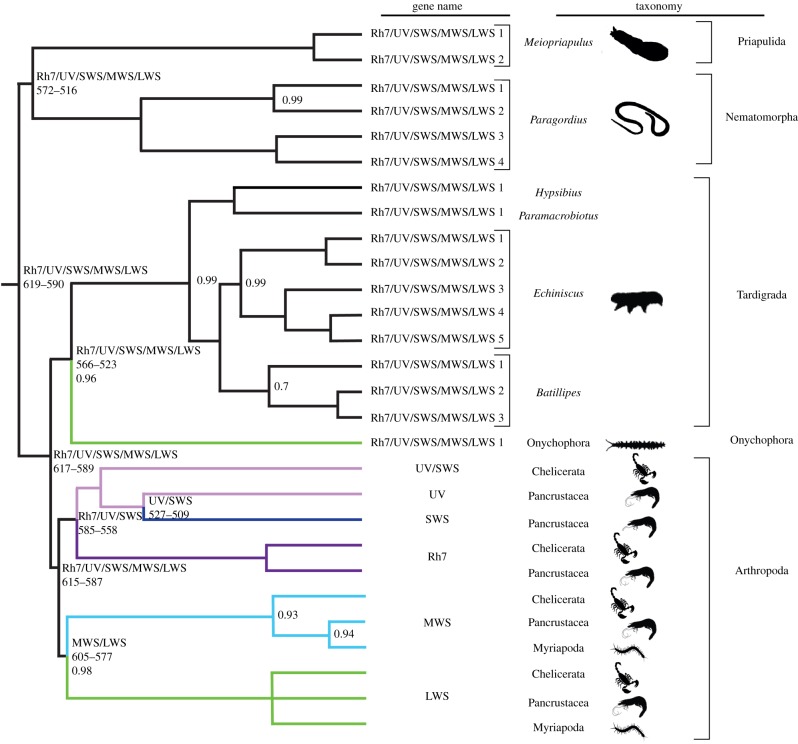
The ecdysozoan visual opsin phylogeny, with a focus on the non-arthropod ecdysozoans. New opsins were identified in *Meiopriapulus*, *Paragordius*, and three species of Tardigrada, showing independent duplications of visual opsins in these lineages. At key internal nodes (top to bottom): the name of opsin (defined according to the rules in §1b) that is inferred to have existed at that node—based on the results of the ALE analysis. Credibility interval for the age of the duplication is given at key nodes within the tree (in Ma)—see electronic supplementary material, figure S6 for the complete set of results. Only posterior probabilities for nodes with a support value lower than 1 are reported, see electronic supplementary material, figure S5 for the complete set of results.

ALE [41] analysis (electronic supplementary material, figure S4), as well as standard visual inspection of the distribution of the opsin paralogues across the species in our ecdysozoan phylogeny, indicate that the new opsins identified in Priapulida, Nematomorpha, and Tardigrada are not orthologous to specific paralogues used by arthropods for colour vision (i.e. they are not homologues of the arthropod's LWS, MWS, UV, Rh7, and SWS opsins). Instead, they represent lineage-specific expansions of the original ecdysozoan visual opsin that we shall hereafter refer to (see §1b above and electronic supplementary material, figure S1) as the Rh7/UV/SWS/LWS/MWS opsin (figure 2), with reference to the named, well-characterized, ecdysozoan visual opsins of which these sequences are orthologues. Within their respective phyla, the new priapulid, nematomorph, and tardigrade opsins could represent family, genus, or even species-specific duplications. Current taxon sampling does not allow us to clarify this problem. In particular, the independent duplications identified by ALE (see electronic supplementary material, figure S4) in the genomes of the tardigrades *Batillipes* sp*.* and *Echiniscus testudo* could be indicative of order-level duplications in Arthrotardigrada and Echiniscoidea, respectively. The large number of opsin paralogues found in *Batillipes* and *Echiniscus* might indicate the possibility that the ability to differentiate between colours independently evolved twice in Tardigrada, and hence multiple times in Ecdysozoa. However, as the expression patterns and wavelengths of maximum absorbance (*λ*_max_) of these novel opsins are unknown, it is not possible at this stage to state with any level of confidence whether colour vision exists outside Arthropoda. Irrespective of whether *Batillipes* and *Echiniscus* can differentiate between colours based on wavelength alone, behavioural studies have demonstrated that these species have a greater ability to react to light [55] when compared to tardigrades such as *Milnesium tardigradum*, that have only one copy of the Rh7/UV/SWS/LWS/MWS opsin [23]. In Priapulida, photic behaviour has not yet been observed, and it is unknown where and for what function the opsins we identified may be expressed [56].

In Arthropoda, only the LWS opsin could be identified in the sea spider (Arthropoda; Chelicerata; Pycnogonida) *Nymphon gracile*, possibly suggesting a process of secondary loss of opsins in this species (figure 2; electronic supplementary material, figure S5). Most myriapod visual opsins cluster in the arthropod LWS clade. The exception to this is a number of sequences restricted only to the Scutigeromorpha (Chilopoda) *Craterostigmus tasmanianus* and *Scutigera coleoptrata* (figure 2; electronic supplementary material, figure S5) that cluster in the MWS clade. The distribution of opsin paralogues across Myriapoda suggests that the molecular component of the visual system of the myriapods for which we have data is degenerate in comparison to the base of their stem lineage. This is consistent with the highly modified eyes observed in crown myriapods [57].

### (c) The history of ecdysozoan vision

**Figure 3.**
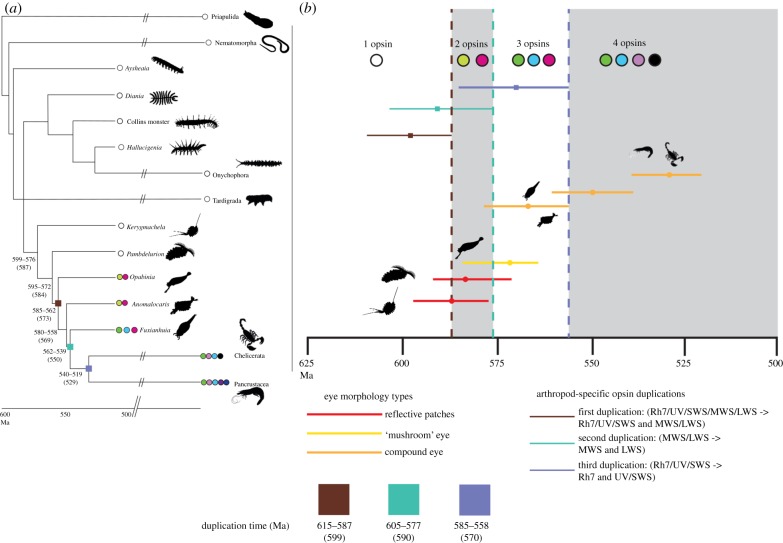
(*a*) Summary of the evolutionary history of the ecdysozoan visual opsins outside crown Arthropoda. An ecdysozoan timetree displaying both fossil and extant forms. Opsins inferred to have existed in terminal taxa are represented as circles. Squares along the tree indicate gene duplications along the arthropod lineage. The colours of the squares identify duplications between panels (*a* and *b*) and do not refer to the *λ*−max of the duplicating gene. Similarly, the colour of the circles indicating the presence of individual opsins at the tips of the tree do not necessarily indicate the *λ*−max of that specific opsin, as such values are not known for all the opsins in the tree. Colours are simply used to identify the presence of specific opsins across taxa. (*b*) Summary scheme concomitantly illustrating the evolution of the molecular and morphological components of the arthropod eye. Top: gene duplication history; gene present at different times and history of gene duplications (average age and 95% credibility intervals). Bottom: age of the nodes representing the separation of stem lineages leading to fossil taxa with specific eye types from the lineage leading to the arthropod last common ancestor. The timescales at the bottom of the figures are in Ma. *Colour codes: opsin genes* (circles in panel *a* and on top of panel *b*). White: Rh7/UV/SWS/MWS/LWS; light green: MWS/LWS; fuchsia: Rh7/UV/SWS; dark green: LWS; light blue: MWS; lilac: Rh7; black: UV/SWS; dark purple: UV; dark blue: SWS. *Opsin duplications* (squares in panel *a* and divergence times credibility bars in panel *b*). Brown: Rh7/UV/SWS/MSW/LWS to Rh7/UV/SWS and MWS/LWS. Teal: MWS/LWS to MWS and LWS. Light purple: Rh7/UV/SWS to Rh7 and UV/SWS. *Age of the stem lineage leading to fossils with specific eye types*. Red: age of the stem lineages leading to *Kerygmachela* and *Pambdelurion* (with reflective patches). Yellow: age of the stem lineage leading to *Opabinia* (with mushroom eyes). Orange: age of the stem lineages leading to *Anomalocaris*, *Fuxianhuia*, and Arthropoda (with compound eyes).

Our ALE [41] analysis (see electronic supplementary material, figure S4) indicates that the last common ecdysozoan ancestor only had one orthologue of the panarthropod visual opsins—the Rh7/UV/SWS/LWS/MWS opsin. ALE analyses confirmed (see also [22,23]) that the Rh7/UV/SWS opsin and the LWS/MWS opsin originated from a duplication of the Rh7/UV/SWS/LWS/MWS opsin that occurred after the separation of both Onychophora and Tardigrada from Arthropoda. The 95% credibility interval from our opsin molecular clock analysis dates this duplication to 615–587 Ma (table 1). Phylogenetic bracketing (figure 3) implies that *Hallucigenia* and other lobopods that are more closely related to Onychophora than they are to Arthropoda (e.g. *Xenusion*, *Microdictyon*, and the Collins Monster) only inherited one orthologue of the panarthropod visual opsins (the Rh7/UV/SWS/LWS/MWS opsin) from their last common ancestor, and possessed the same opsin complement as the extant Onychophora. Unless lineage-specific duplications occurred along the lineages leading to these taxa, they could have only expressed one visual opsin. Fossil evidence indicates that the eyes of these taxa were similar to those of the Onychophora [14], suggesting that, like the Onychophora, these taxa were most likely monochromats.

**Table 1.** Identity, age of duplication, function, and distribution of the ecdysozoan visual opsins.

name	age of duplication (Ma)	function where extant	taxonomic distribution
Rh7/UV/SWS/MWS/LWS	n.a.	MWS [22]	Priapulida, Nematomorpha, Tardigrada, *Kerygmachela*, *Pambdelurion*
Rh7/UV/SWS	615–587	n.a.	*Opabinia*, *Anomalocaris*, *Fuxianhuia*
MWS/LWS	615–587	n.a.	*Opabinia*, *Anomalocaris*
MWS	605–577	MWS	*Fuxianhuia*, Chelicerata, Pancrustacea
LWS	605–577	LWS	*Fuxianhuia*, Chelicerata, Pancrustacea
UV/SWS	585–558	UV	Chelicerata
Rh7	585–558	UV (non-visual)	Chelicerata, Pancrustacea
UV	527–509	UV	Pancrustacea
SWS	527–509	UV	Pancrustacea

Results from ALE [41] and an inspection of our opsin phylogeny clearly indicated that the last common ancestor of the crown arthropods (i.e. the last common ancestor of Mandibulata and Chelicerata) possessed four opsins—the UV/SWS opsin, and the Rh7, MWS, and LWS opsins (figure 2; electronic supplementary material, figures S5 and S6). The iconic Cambrian trilobites, which are currently interpreted as members of crown Arthropoda (either representing a stem chelicerate or a stem mandibulate lineage [24,58]), must have inherited, at the base of their stem lineage, four opsins—the UV/SWS opsin, Rh7, MWS, and LWS. Phylogenetic bracketing further implies that trilobites could not have possessed both UV and SWS opsins as these paralogues emerged from a duplication of the UV/SWS opsin that occurred more crownward in the arthropod tree, within Pancrustacea (see also above). The four opsins inherited at the base of the total group Trilobita constitute the most likely opsin complement for the trilobite last common ancestor, which minimally should have therefore been capable of trichromatic colour vision (assuming Rh7 was used for circadian entrainment [20]). However, we cannot rule out the possibility of lineage-specific opsin deletions either along the stem trilobite lineage or within crown Trilobita—e.g. in the eyeless *Trimerocephalus* [59]. Similarly, lineage-specific duplications may have occurred along the stem trilobite lineage or in more visually acute forms, much like the lineage-specific multiple duplications that occurred in Stomatopoda [60].

Deducing the likely opsin complement of individual stem arthropod taxa is more complex. This paraphyletic lineage includes the earliest apex predators of the Cambrian, such as the anomalocaridids, but the extant taxa that bracket the group possess one and four opsins, respectively (see figure 2 and above). This leaves open the possibility of the existence of intermediate states in these fossils, the genomes of which cannot be sequenced. We compared divergence times for the opsin genes across the arthropod stem lineage to estimate the likely opsin complement inherited by specific fossil taxa.

The duplication separating the Rh7/UV/SWS opsin from the LWS/MWS opsin occurred between 615 and 587 Ma (table 1, figure 3*a*; electronic supplementary material, figure S6). According to our total evidence, arthropod divergence time analysis, this postdates the split between *Pambdelurion* and all remaining arthropods (595–572 Ma). Yet, it predates the divergence between *Opabinia* and the remaining arthropod lineages (585–562 Ma; figure 3*a* and *b*, electronic supplementary material, figure S7). We thus infer that two opsin genes (the Rh7/UV/SWS opsin and the LWS/MWS opsin) were inherited at the base of the stem lineage leading to *Opabinia*. If no lineage-specific deletion happened along this lineage, *Opabinia* could have been capable of dichromatic colour vision. The absorbance spectra of the Rh7/UV/SWS opsin and the LWS/MWS opsin are unknown. However, stem arthropods are known from relatively shallow waters where UV wavelengths would have provided high levels of contrast between objects and their backgrounds [61]. Hence, we conjecture that the Rh7/UV/SWS opsin might have been UV sensitive. Large sediment influx in shallow waters due to a lack of rooting in terrestrial environments would have also made the LWS pigment useful for behavioural tasks [62], and we conjecture that the LWS/MWS opsin might have been LW sensitive. Both hypotheses could be tested using ancestral protein resurrection techniques (e.g. [19]), but this is outside the scope of our study. From a morphological perspective, it is worth noting that while the anatomical features of the eye of the last common ancestor of *Opabinia* and of the remaining arthropods are unknown, *Opabinia* itself displays five mushroom-type compound eyes [63] that are much more complex than the reflective eye patches of *Kerygmachela* [64] and *Pambdelurion* (see electronic supplementary material and electronic supplementary material, figure S8), both of which, according to our analyses, possessed only one opsin gene—the Rh7/UV/SWS/LWS/MWS Opsin.

Our ALE analyses and our opsin timetree (figures 2 and 3) indicate that the next duplication along the stem arthropod lineage was that of the LWS/MWS Opsin. This duplication, which we dated to 605–577 Ma, resulted in the origin of the LWS and the MWS opsins. A comparison of the age of the LWS/MWS Opsin duplication against our arthropod divergence times indicates that this duplication postdated the divergence of *Anomalocaris* from the remaining stem and crown arthropods (580–558 Ma), but predated the split between *Fuxianhuia* and the crown arthropods (562–539 Ma). From these results we infer that, like *Opabinia*, the base of the stem lineage leading to *Anomalocaris* also inherited two opsin genes (the Rh7/UV/SWS Opsin and the LWS/MWS Opsin), while the base of the stem lineage leading to *Fuxianhuia* may have inherited three genes (the Rh7/UV/SWS Opsin, the LWS, and the MWS opsins). These inferences suggest that while *Anomalocaris* was dichromatic at best, *Fuxianhuia* might have been capable of trichromatic colour vision. From a morphological perspective, *Anomalocaris* possesses unique, pear-shaped, compound eyes with a number of lenses comparable to that of modern arthropods [65,66], while *Fuxianhuia* possessed a compound eye comparable in resolution to those of modern malacostracans (figure 3) [51].

The duplication of the Rh7/UV/SWS Opsin led to the evolution of the Rh7 and UV/SWS Opsin. We date this duplication to 585–558 Ma, postdating the split between *Fuxianhuia* and the crown arthropods (562–539 Ma), but predating the split between Mandibulata and Chelicerata (540–519 Ma) (table 1). This agrees with observations on living arthropods that always minimally possess four opsins (LWS, MWS, Rh7, and the UV-sensitive UV/SWS Opsin) unless they have undergone lineage-specific deletions (as in the case of the myriapods and possibly *Nymphon gracile*). Finally, a duplication of the UV/SWS Opsin, that we date at 527–509 Ma, resulted in the evolution of the UV and SWS opsins, that should be limited to the taxa descending from the last common ancestor of the Allotriocarida [21] (where these opsins are known in all lineages with eyes—Branchiopoda and Hexapoda) and Malacostraca.

## 4. Conclusion

A combination of molecular and palaeontological information can be used to improve our understanding of the physiological capabilities of extinct animals. This approach has been demonstrated here with reference to the Cambrian ecdysozoans, where we demonstrated polychromatism for the trilobites based on phylogenetic bracketing and inferred the existence of a variety of stem arthropod lineages capable of dichromatic and trichromatic vision. New and diverse body plans emerge in the Cambrian, implying a drive for different lifestyles. These are reflected in diverse visual systems [14,15,51,63–65,67]. We show a shared narrative where extinct taxa with more complex eyes are also predicted to have more opsin genes, suggesting that morphological and molecular changes in the ecdysozoan visual systems were, at least to some extent, coupled and demonstrate the power of combining molecular and palaeontological data in a molecular palaeobiological approach to the study of the evolution of life.

## Supplementary Material

Supplemental Figures 1-8

## Supplementary Material

Supplemental Methods

## Data accessibility

All data gathered and analysed within this project is publicly available, alongside all of our analyses, at: https://bitbucket.org/bzxdp/fleming_ecdysozoan_opsin_evolution/src. Accession numbers for sequences uploaded to GenBank are available in the electronic supplementary material.

## References

[1] Parker AR 1998 Colour in Burgess Shale animals and the effect of light on evolution in the Cambrian. Proc. R. Soc. Lond. B 265**,** 967–972. (10.1098/rspb.1998.0385)

[2] Vinther J, Stein M, Longrich NR, Harper DA 2014 A suspension-feeding anomalocarid from the Early Cambrian. Nature 507**,** 496–499. (10.1038/nature13010) 24670770

[3] Marshall CR 2006 Explaining the Cambrian ‘explosion’ of animals. Annu. Rev. Earth Planet. Sci. 34**,** 355–384. (10.1146/annurev.earth.33.031504.103001)

[4] Erwin DH, Valentine JW 2012 The Cambrian explosion: the construction of animal biodiversity. Greenwood Village, CO: Roberts & Company.

[5] Owens GL, Rennison DJ 2017 Evolutionary ecology of opsin gene sequence, expression and repertoire. Mol. Ecol. 26**,** 1207–1210. (10.1111/mec.14032) 28271616

[6] Paul KN, Saafir TB, Tosini G 2009 The role of retinal photoreceptors in the regulation of circadian rhythms. Rev. Endocr. Metab. Disord. 10**,** 271–278. (10.1007/s11154-009-9120-x) 19777353 PMC2848671

[7] Strausfeld NJ, Ma X, Edgecombe GD, Fortey RA, Land MF, Liu Y, Cong P, Hou X 2016 Arthropod eyes: the early Cambrian fossil record and divergent evolution of visual systems. Arthropod Struct. Dev. 45**,** 152–172. (10.1016/j.asd.2015.07.005) 26276096

[8] Nilsson D-E 2009 The evolution of eyes and visually guided behaviour. Phil. Trans. R. Soc. B 364**,** 2833–2847. (10.1098/rstb.2009.0083) 19720648 PMC2781862

[9] Vannier J, Liu J, Lerosey-Aubril R, Vinther J, Daley AC 2014 Sophisticated digestive systems in early arthropods. Nat. Commun. 5**,** 3641 (10.1038/ncomms4641) 24785191

[10] Butterfield NJ 2003 Exceptional fossil preservation and the Cambrian explosion. Integr. Comp. Biol. 43**,** 166–177. (10.1093/icb/43.1.166) 21680421

[11] Vinther J, Porras L, Young FJ, Budd GE, Edgecombe GD 2016 The mouth apparatus of the Cambrian gilled lobopodian *Pambdelurion whittingtoni*. Palaeontology 59**,** 841–849. (10.1111/pala.12256)

[12] Daley AC, Bergström J 2012 The oral cone of *Anomalocaris* is not a classic ‘peytoia’. Naturwissenschaften 99**,** 501–504. (10.1007/s00114-012-0910-8) 22476406

[13] Waloszek D, Maas A, Chen J, Stein M 2007 Evolution of cephalic feeding structures and the phylogeny of Arthropoda. Palaeogeogr. Palaeoclimatol. Palaeoecol. 254**,** 273–287. (10.1016/j.palaeo.2007.03.027)

[14] Ma X, Hou X, Edgecombe GD, Strausfeld NJ 2012 Complex brain and optic lobes in an early Cambrian arthropod. Nature 490**,** 258–261. (10.1038/nature11495) 23060195

[15] Tanaka G, Hou X, Ma X, Edgecombe GD, Strausfeld NJ 2013 Chelicerate neural ground pattern in a Cambrian great appendage arthropod. Nature 502**,** 364 (10.1038/nature12520) 24132294

[16] Daley AC, Paterson JR, Edgecombe GD, García-Bellido DC, Jago JB 2013 New anatomical information on *Anomalocaris* from the Cambrian Emu Bay Shale of South Australia and a reassessment of its inferred predatory habits. Palaeontology 56**,** 971–990.

[17] Terakita A 2005 The opsins. Genome Biol. 6**,** 213 (10.1186/gb-2005-6-3-213) 15774036 PMC1088937

[18] Henze MJ, Oakley TH 2015 The dynamic evolutionary history of pancrustacean eyes and opsins. Integr. Comp. Biol. 55**,** 830–842. (10.1093/icb/icv100) 26319405

[19] Dungan SZ, Kosyakov A, Chang BS 2015 Spectral tuning of killer whale (*Orcinus orca*) rhodopsin: evidence for positive selection and functional adaptation in a cetacean visual pigment. Mol. Biol. Evol. 33**,** 323–336. (10.1093/molbev/msv217) 26486871

[20] Ni JD, Baik LS, Holmes TC, Montell C 2017 A rhodopsin in the brain functions in circadian photoentrainment in *Drosophila*. Nature 545**,** 340–344. (10.1038/nature22325) 28489826 PMC5476302

[21] Oakley TH, Wolfe JM, Lindgren AR, Zaharoff AK 2012 Phylotranscriptomics to bring the understudied into the fold: monophyletic Ostracoda, fossil placement, and pancrustacean phylogeny. Mol. Biol. Evol. 30**,** 215–233. (10.1093/molbev/mss216) 22977117

[22] Hering L et al. 2012 Opsins in Onychophora (velvet worms) suggest a single origin and subsequent diversification of visual pigments in arthropods. Mol. Biol. Evol. 29**,** 3451–3458. (10.1093/molbev/mss148) 22683812

[23] Hering L, Mayer G 2014 Analysis of the opsin repertoire in the tardigrade *Hypsibius dujardini* provides insights into the evolution of opsin genes in Panarthropoda. Genome Biol. Evol. 6**,** 2380–2391. (10.1093/gbe/evu193) 25193307 PMC4202329

[24] Rota-Stabelli O, Daley AC, Pisani D 2013 Molecular timetrees reveal a Cambrian colonization of land and a new scenario for ecdysozoan evolution. Curr. Biol. 23**,** 392–398. (10.1016/j.cub.2013.01.026) 23375891

[25] Lozano-Fernandez J et al. 2016 A molecular palaeobiological exploration of arthropod terrestrialization. Phil. Trans. R. Soc. B 371, 20150133 (10.1098/rstb.2015.0133) 27325830 PMC4920334

[26] Schwentner M, Richter S, Rogers DC, Giribet G 2018 Tetraconatan phylogeny with special focus on Malacostraca and Branchiopoda: highlighting the strength of taxon-specific matrices in phylogenomics. Proc. R. Soc. B 285, 20181524 (10.1098/rspb.2018.1524) PMC6125901 30135168

[27] Wolfe JM, Daley AC, Legg DA, Edgecombe GD 2016 Fossil calibrations for the arthropod Tree of Life*.* Earth Sci. Rev. 160, 43–110. (10.1016/j.earscirev.2016.06.008)

[28] Zhang XG, Siveter DJ, Waloszek D, Maas A 2007 An epipodite-bearing crown-group crustacean from the Lower Cambrian. Nature 449**,** 595 (10.1038/nature06138) 17914395

[29] Zhong YF, Butts T, Holland PW 2008 HomeoDB: a database of homeobox gene diversity. Evol. Dev. 10**,** 516–518. (10.1111/j.1525-142X.2008.00266.x) 18803769

[30] Haft DH, Loftus BJ, Richardson DL, Yang F, Eisen JA, Paulsen IT, White O 2001 TIGRFAMs: a protein family resource for the functional identification of proteins. Nucleic Acids Res. 29**,** 41–43. (10.1093/nar/29.1.41) 11125044 PMC29844

[31] Camacho C, Coulouris G, Avagyan V, Ma N, Papadopoulos J, Bealer K, Madden TL 2009 BLAST+: architecture and applications. BMC Bioinformatics 10**,** 421 (10.1186/1471-2105-10-421) 20003500 PMC2803857

[32] Feuda R, Hamilton SC, McInerney JO, Pisani D 2012 Metazoan opsin evolution reveals a simple route to animal vision. Proc. Natl Acad. Sci. USA 109**,** 18 868–18 872. (10.1073/pnas.1204609109) 23112152 PMC3503164

[33] Feuda R, Rota-Stabelli O, Oakley TH, Pisani D 2014 The comb jelly opsins and the origins of animal phototransduction. Genome Biol. Evol. 6**,** 1964–1971. (10.1093/gbe/evu154) 25062921 PMC4159004

[34] Ramirez MD, Pairett AN, Pankey MS, Serb JM, Speiser DI, Swafford AJ, Oakley TH 2016 The last common ancestor of most bilaterian animals possessed at least nine opsins. Genome Biol. Evol. 8**,** 3640–3652. 28172965 10.1093/gbe/evw248 PMC5521729

[35] Edgar RC 2004 MUSCLE: multiple sequence alignment with high accuracy and high throughput. Nucleic Acids Res. 32**,** 1792–1797. (10.1093/nar/gkh340) 15034147 PMC390337

[36] Löytynoja A 2014 Phylogeny-aware alignment with PRANK. Methods Mol. Biol. 1079**,** 155–170. (10.1007/978-1-62703-646-7_10) 24170401

[37] Lartillot N, Lepage T, Blanquart S 2009 PhyloBayes 3: a Bayesian software package for phylogenetic reconstruction and molecular dating. Bioinformatics 25**,** 2286–2288. (10.1093/bioinformatics/btp368) 19535536

[38] Fleming JFF, Møbjerg Kristensen R, Vinther Sørensen M, Park T-YS, Arakawa K, Blaxter M, Rebecchi L, Guidetti R, Williams TA, Roberts NW, Vinther J, Pisani D 2018 Data From: Molecular palaeontology illuminates the evolution of ecdysozoan vision *Dryad Digital Repository*. (http://doi:10.5061/dryad.ts895r1) 10.1098/rspb.2018.2180 PMC6283943 30518575

[39] Abascal F, Posada D, Zardoya R 2006 MtArt: a new model of amino acid replacement for Arthropoda. Mol. Biol. Evol. 24**,** 1–5. (10.1093/molbev/msl136) 17043087

[40] Yang Z 2007 PAML 4: phylogenetic analysis by maximum likelihood. Mol. Biol. Evol. 24**,** 1586–1591. (10.1093/molbev/msm088) 17483113

[41] Szöllősi GJ, Rosikiewicz W, Boussau B, Tannier E, Daubin V 2013 Efficient exploration of the space of reconciled gene trees. Syst. Biol. 62**,** 901–912. (10.1093/sysbio/syt054) 23925510 PMC3797637

[42] Lepage T, Bryant D, Philippe H, Lartillot N 2007 A general comparison of relaxed molecular clock models. Mol. Biol. Evol. 24, 2669–2680. (10.1093/molbev/msm193) 17890241

[43] Yuan X, Chen Z, Xiao S, Zhou C, Hua H 2011 An early Ediacaran assemblage of macroscopic and morphologically differentiated eukaryotes. Nature 470**,** 390 (10.1038/nature09810) 21331041

[44] Benton MJ 2015 Exploring macroevolution using modern and fossil data. Proc. R. Soc. B 282**,** 20150569 (10.1098/rspb.2015.0569) PMC4590474 26063844

[45] Benton MJ, Donoghue PCJ, Asher RJ 2009 Calibrating and constraining molecular clocks. In The timetree of life (eds Hedges SB, Kumar S), pp. 35–86. Oxford, UK: Oxford University Press.

[46] Vannier J, Calandra I, Gaillard C, Zylinska A 2010 Priapulid worms: pioneer horizontal burrowers at the Precambrian-Cambrian boundary. Geology 38**,** 711–714. (10.1130/G30829.1)

[47] Zhang C, Stadler T, Klopfstein S, Heath TA, Ronquist F 2015 Total-evidence dating under the fossilized birth–death process. Syst. Biol. 65**,** 228–249. (10.1093/sysbio/syv080) 26493827 PMC4748749

[48] Heath TA, Huelsenbeck JP, Stadler T 2014 The fossilized birth–death process for coherent calibration of divergence-time estimates. Proc. Natl Acad. Sci. USA 111**,** E2957–E2966. (10.1073/pnas.1319091111) 25009181 PMC4115571

[49] Höhna S, Heath TA, Boussau B, Landis MJ, Ronquist F, Huelsenbeck JP 2014 Probabilistic graphical model representation in phylogenetics. Syst. Biol. 63**,** 753–771. (10.1093/sysbio/syu039) 24951559 PMC4184382

[50] Höhna S, Landis MJ, Heath TA, Boussau B, Lartillot N, Moore BR, Huelsenbeck JP, Ronquist F 2016 RevBayes: Bayesian phylogenetic inference using graphical models and an interactive model-specification language. Syst. Biol. 65**,** 726–736. (10.1093/sysbio/syw021) 27235697 PMC4911942

[51] Yang J, Ortega-Hernández J, Butterfield NJ, Liu Y, Boyan GS, Hou J, Lan T, Zhang X 2016 Fuxianhuiid ventral nerve cord and early nervous system evolution in Panarthropoda. Proc. Natl Acad. Sci. USA 113**,** 2988–2993. (10.1073/pnas.1522434113) 26933218 PMC4801254

[52] Legg DA, Sutton MD, Edgecombe GD 2013 Arthropod fossil data increase congruence of morphological and molecular phylogenies. Nat. Commun. 4**,** 2485 (10.1038/ncomms3485) 24077329

[53] Ødegaard F 2000 How many species of arthropods? Erwin's estimate revised. Biol. J. Linn. Soc. 71, 583–597. (10.1111/j.1095-8312.2000.tb01279.x)

[54] O'Reilly JE, dos Reis M, Donoghue PC 2015 Dating tips for divergence-time estimation. Trends Genet. 31, 637–650. (10.1016/j.tig.2015.08.001) 26439502

[55] Shcherbakov D, Schill RO, Brümmer F, Blum M 2010 Movement behaviour and video tracking of *Milnesium tardigradum* Doyère, 1840 (Eutardigrada, Apochela). Contrib. Zool. 79, 33–38.

[56] Schmidt-Rhaesa A 2013 *Priapulida* In Handbook of zoology Vol 1: Nematomorpha, Priapulida, Kinorhyncha, Loricifera (ed. Schmidt-Rhaesa A), pp. 147–180. Berlin, Germany: Walter De Gruyter.

[57] Harzsch S, Melzer RR, Müller CH 2007 Mechanisms of eye development and evolution of the arthropod visual system: the lateral eyes of Myriapoda are not modified insect ommatidia. Org. Divers. Evol. 7, 20–32. (10.1016/j.ode.2006.02.004)

[58] Scholtz G, Edgecombe GD 2005 Heads, Hox and the phylogenetic position of trilobites. In Crustacean Issues Vol. 16, Crustacea and Arthropod Relationships (eds Koenemann F, Jenner RA). CRC Press, Boca Raton, USA.

[59] Błażejowski B, Brett CE, Kin A, Radwański A, Gruszczyński M 2016 Ancient animal migration: a case study of eyeless, dimorphic Devonian trilobites from Poland. Palaeontology 59**,** 743–751. (10.1111/pala.12252)

[60] Porter ML, Bok MJ, Robinson PR, Cronin TW 2009 Molecular diversity of visual pigments in Stomatopoda (Crustacea). Vis. Neurosci. 26**,** 255–265. (10.1017/S0952523809090129) 19534844

[61] Helfman G, Collette BB, Facey DE, Bowen BW 2009 The diversity of fishes: biology, evolution, and ecology. Chichester, UK: John Wiley & Sons.

[62] Davies NS, Gibling MR 2010 Cambrian to Devonian evolution of alluvial systems: the sedimentological impact of the earliest land plants. Earth Sci. Rev. 98**,** 171–200. (10.1016/j.earscirev.2009.11.002)

[63] Whittington HB 1975 The enigmatic animal *Opabinia regalis*, Middle Cambrian, Burgess Shale, British Columbia. Phil. Trans. R. Soc. Lond. B 271**,** 1–43. (10.1098/rstb.1975.0033) PMC4360120 25750235

[64] Park T-YS et al. 2018 Brain and eyes of *Kerygmachela* reveal protocerebral ancestry of the panarthropod head. Nat. Commun. 9, 1019. 29523785 10.1038/s41467-018-03464-w PMC5844904

[65] Paterson JR, Garcia-Bellido DC, Lee MS, Brock GA, Jago JB, Edgecombe GD 2011 Acute vision in the giant Cambrian predator Anomalocaris and the origin of compound eyes. Nature 480**,** 237–240. (10.1038/nature10689) 22158247

[66] Daley AC, Edgecombe GD 2014 Morphology of *Anomalocaris canadensis* from the Burgess *Shale**.* J. Paleontol. 88**,** 68–91. (10.1666/13-067)

[67] Schoenemann B, Liu JN, Shu DG, Han J, Zhang ZF 2009 A miniscule optimized visual system in the Lower Cambrian. Lethaia 42**,** 265–273. (10.1111/j.1502-3931.2008.00138.x)

