# Supplementary material for: Molecular palaeontology illuminates the evolution of ecdysozoan vision
Source: Proc Biol Sci. 2018 Dec 5;285(1892):20182180. doi: 10.1098/rspb.2018.2180 (PMC6283943; doi:10.1098/rspb.2018.2180)
Supplement: Supplemental Figures 1-8 [file rspb20182180supp1.pdf]

Naming Opsins

Describing the evolutionary history of a gene family is complex when, as in the case of the Opsin family, no clear nomenclature rule has consistently been applied to name newly identified genes. Here, to allow for a meaningful discussion of the evolutionary history of the Opsin family, in terms of gene deletions and duplications, we shall follow well-established rules from the regulatory gene literature (as applied for example in [27, 28]). This gene naming strategy prescribe that if Gene A is present in single copy in Lineage φ (where it is called Gene A1) but it is present in 2 copies in Lineage ψ (where the two paralogs are called Genes A1 and A2), Gene A1 from lineage φ, which is orthologous to both Genes A1 and A2 in Lineage ψ, should be referred to as Gene A1/A2 (see [28] for an exemplar classic paper and [27] for a well-known database implementing using this gene naming convention) and see Supplemental Figure 1 for a schematic representation. To illustrate this approach practically, we shall use the example of the evolutionary relations between the UV and SWS opsins discussed in main text. In the Opsin literature, in all arthropods that do not have distinguished UV and SWS opsins (for example the spiders), the single copy orthologue of the UV and SWS genes is referred to as the "UV opsin" based on functional considerations. In an evolutionary framework, this functional naming scheme can be easily overinterpreted as implying that it was an ancestral UV gene that duplicated, with the SWS gene neofunctionalising after duplication. While this is a valid hypothesis, such an inference is unwarranted because the process of duplication has no functional memory. Accordingly, it might well have been that the "UV gene" underwent neofunctionalization (turning into a functionally SWS gene) in the stem pancrustacean lineage, before duplication. If that were the case, it would have been the gene that in malacostracans and allotriocarids is functionally short-wave sensitive (i.e. the SWS gene) that duplicated with the allitriocarid and malacostracan UV gene being those that emerged from a neofunctionalization process. Here, by naming the spider ortholog of the allotriocarid and malacostracan UV and SWS genes, the "UV/SWS Opsin", rather than the "UV Opsin" we will simply limit ourselves to stating that the UV and SWS Opsins emerged from a duplication of their last

common ancestor, which is correct irrespective of the functions of the UV and SWS opsins ancestor. This gene nomenclature strategy allows for an unambiguous identification of genes along a tree and a precise description of evolutionary processes underpinned by gene duplications (see Supplemental Figure 1c and d for a practical example). However, this approach is not flawless as it is ambiguous with reference to the function of the genes to which composite names are assigned. To counter this problem, when the function of a gene with a composite name is known, we shall point this out explicitly. For example, we shall refer to the spiders' UV/SWS Opsin – which is UV-sensitive, as the "UV sensitive UV/SWS Opsin". We do not intend these names to be permanent designations for these opsin genes, but rather we intend to optimise the discussion of gene duplications in the family without defining new names for clades that might be reasonably overturned in future analyses.

Table 1: SRA accession numbers for genomes and transcriptomes in this manuscript. Bold indicates entries sequenced for this project

Phylum	Taxa	Order	SRA or Bioproject Number (If Any)	Opsin Number
Hexapoda	*Baetis_sp*	Ephemeroptera	SRR921569	5
	Boreus_hyemalis	Mecoptera	SRR921574	6
	Corydalus_cornutus	Megaloptera	SRR921584	3
	Empusa_pennata	Mantodea	SRR921590	2
	Fopius_arisanus	Hymenoptera	SRR1560653	3
	Haploembia_palaui	Embioptera	SRR921605	2
	Liposcelis_entomophila	Psocoptera	SRR950092	2
	Meinertellus_cundinamarcensis	Archaeognatha	SRR921617	1
	Menopon_gallinae	Phthiraptera	SRR921619	1
	Periplaneta_americana	Blattodea	SRR921630	1
	Stylops_melittae	Strepsiptera	SRR921643	1
Myriapoda	*Abacion_magnum*	Callipodida	SRX326781	1
	Brachycybe lecontii	Platydesmida	SRX326776	0
	Cambala_annulata	Spirostreptida	SRX326783	1
	Cleidogona_sp	Chordeumatida	SRX326780	1
	Craterostigmus tasmanianus	Craterostigmimorpha	SRR1157986	1
	Eupolybothrus_cavernicolus	Lithobiomorpha	ERX311347	1
	Petaserpes sp.	Polyzoniida	SRX326777	0
	Prostemmiulus_sp	Stemmiulida	SRX326782	1
	Scutigera_coleoptrata	Scutigeramorpha	SRR1158078	1
Isopoda	***Oniscus_sp***	Oniscidea	PRJNA438779	1
Chelicerata	*Centruroides sp.*	Scorpiones	SRX451012	0
	Damon_sp	Amblypygi	PRJNA438779	1

Phylum	Taxa	Order	SRA or Bioproject or Accession Number	Opsin Number
	Frontinella sp.	Araneae	SRX451004	0
	Galeodes_sp	Solifugae	PRJNA438779	1
	Limulus_polyphemus	Xiphosura	PRJNA438779	2
	Liphistius sp.	Araneae	SRX652489	0
	Neobisium carcinoides	Pseudoscorpionida	PRJNA438779	0
	Neoscona arabesca	Araneae	SRX451007	0
	Nymphon gracile	Pycnogonida	PRJNA438779	1
	Oligolophus sp.	Opiliones	PRJNA438779	1
Priapulida	***Meiopriapulus_fijiensis***	Priapulimorpha	PRJNA438779	2
	Tubiluchus sp.	Priapulimorpha	SRX1122235	0
Nematomorpha	*Paragordius_varius*	Gordioidea	ERP021314	4
Tardigrada	***Batillipes_sp***	Arthrotardigrada	MH550105, MH550106, MH550107	3
	Echiniscus_testudo	Echiniscoidea	MH550108, MH550109, MH550110, MH550111, MH550112	5
	Paramacrobiotus_richtersi	Parachela	MH550113	1
	Ramazzottius varieornatus	Parachela	PRJDB5471	0

Table 2: The sequence name and species name of each opsin in the final alignment

Sequence Name	Species_Name
Abacion_Abacion_clean_fasc4895	*Abacion_magnum*
XP_001944926_Rh7_likeA_Acyrtho	*Acyrthosiphon_pisum*
XP_001943275_Rh7_likeB_Acyrtho	*Acyrthosiphon_pisum*
XP_001947730_LW_Acyrthosiphon_	*Acyrthosiphon_pisum*
XP_001951588_UV_A_Acyrthosipho	*Acyrthosiphon_pisum*
XP_001951613_UV_B_Acyrthosipho	*Acyrthosiphon_pisum*
XP_308329_AGAP007548_PB_GPROP1	*Anopheles_gambiae*
XP_003435763_AGAP013149_PA_GPR	*Anopheles_gambiae*
XP_001238571_AGAP001162_PA_GPR	*Anopheles_gambiae*
XP_312478_AGAP002462_PA_GPROP7	*Anopheles_gambiae*
XP_319247_AGAP010089_PA_GPROP9	*Anopheles_gambiae*
Pycnogonid_genome1	*Anoplydactylus_sp*
AAA69069_AmLop1_Apis_mellifera	*Apis_mellifera*
NP_001071293_AmLop2_Apis_melli	*Apis_mellifera*
AAC13418_AmUVop_Apis_mellifera	*Apis_mellifera*
AAC13417_AmBLop_Apis_mellifera	*Apis_mellifera*
ABI48867_opsin_c1_Archaeomysis	*Archaeomysis_grebnitzkii*
Ephemeroptera6	*Baetis_sp*
Ephemeroptera1	*Baetis_sp*
Ephemeroptera3	*Baetis_sp*
Ephemeroptera2	*Baetis_sp*
Ephemeroptera4	*Baetis_sp*
Batillipes2_scf7180001269234_O	*Batillipes_sp*
Batillipes3_scf7180001253643_O	*Batillipes_sp*
Batillipes1_scf7180001269268_N	*Batillipes_sp*
BGIBMGA012539_PA_silkdb_org_UN	*Bombyx_mori*
BGIBMGA007787_PA_silkdb_org_Lo	*Bombyx_mori*
NP_001036882_Lop2_LW_opsin_cer	*Bombyx_mori*
Mecoptera1	*Boreus_hyemalis*
Mecoptera3	*Boreus_hyemalis*
Mecoptera4	*Boreus_hyemalis*
Mecoptera5	*Boreus_hyemalis*
Mecoptera2	*Boreus_hyemalis*
Mecoptera6	*Boreus_hyemalis*
BAG80984_opsin_RhA_Branchinell	*Branchinella_kugenumaensis*
BAG80985_opsin_RhC_Branchinell	*Branchinella_kugenumaensis*
BAG80986_opsin_RhD_Branchinell	*Branchinella_kugenumaensis*
BAG80987_opsin_Rhb_clade_c1_11	*Branchinella_kugenumaensis*
BAG80989_opsin_Rhb_clade_c4_11	*Branchinella_kugenumaensis*
Cambala_Cambala_clean_fasc1729	*Cambala_annulata*
AAB97666_rhodopsin_Cambarellus	*Cambarellus_shufeldtii*
AAC47083_rhodopsin_Camponotus_	*Camponotus_abdominalis*

AAC05092_SW_opsin_Camponotus_a	*Camponotus_abdominalis*
Cleidogona_2	*Cleidogona_sp*
Megaloptera1	*Corydalus_cornutus*
Megaloptera3	*Corydalus_cornutus*
Megaloptera2	*Corydalus_cornutus*
Ctasmianus_Ctasmianus_fasc3868	*Craterostigmus_tasmianus*
CCO61974_rhodopsin_2_Cupienniu	*Cupiennius_salei*
CCO61973_rhodopsin_1_Cupienniu	*Cupiennius_salei*
CCO61975_rhodopsin_3_Cupienniu	*Cupiennius_salei*
Amblypygi1	*Damon_sp*
EFX70801_UNOP1_DAPPUDRAFT_3469	*Daphnia_pulex*
EFX70796_UNOP2_DAPPUDRAFT_3469	*Daphnia_pulex*
EFX75461_blue_wavelength_opsin	*Daphnia_pulex*
EFX81332_UV_wavelength_opsin_D	*Daphnia_pulex*
EFX77537_LOPB1_LOPB_clade_1_15	*Daphnia_pulex*
EFX77473_LOPB5_LOPB_clade_1_15	*Daphnia_pulex*
EFX66668_LOPA6_LOPA_cladeII_6_	*Daphnia_pulex*
EFX63132_LOPA10_LOPA_cladeII_6	*Daphnia_pulex*
EFX63568_LOPA1_LOPA_cladeI_1_4	*Daphnia_pulex*
EFX63570_LOPA3_LOPA_cladeI_1_4	*Daphnia_pulex*
ACN39591_LW_opsin_Dianemobius_	*Dianemobius_nigrofasciatus*
BAG71429_UV_opsin_Dianemobius_	*Dianemobius_nigrofasciatus*
BAF45422_blue_opsin_Dianemobiu	*Dianemobius_nigrofasciatus*
AAF49949_Rh7_Drosophila_melano	*Drosophila_melanogaster*
CAB06821_Rh6_Drosophila_melano	*Drosophila_melanogaster*
AAA28734_Rh2_Drosophila_melano	*Drosophila_melanogaster*
AAA28733_Rh1_Drosophila_melano	*Drosophila_melanogaster*
AAA28854_Rh3_Drosophila_melano	*Drosophila_melanogaster*
NP_476701_Rh4_Drosophila_melan	*Drosophila_melanogaster*
AAC47426_Rh5_Drosophila_melano	*Drosophila_melanogaster*
Echiniscus_7_jcf7180001734070_	*Echiniscus_sp*
Echiniscus_3_jcf7180001530396_	*Echiniscus_sp*
Echiniscus_1_jcf7180001548138_	*Echiniscus_sp*
Echiniscus_6_jcf7180001550879_	*Echiniscus_sp*
Echiniscus4_jcf7180001533989_O	*Echiniscus_sp*
Mantodea2	*Empusa_pennata*
Mantodea1	*Empusa_pennata*
AFM43711_onychopsin_Eoperipatu	*Eoperipatus_sp*
AFM75825_onychopsin_Epiperipat	*Epiperipatus_isthmicola*
CCP46946_onychopsin_Euperipato	*Euperipatoides_kanangrensis*
AFM75824_onychopsin_Euperipato	*Euperipatoides_rowelli*
ABI48870_opsin_c2_Euphausia_su	*Euphausia_superba*
Eupolybothrus_Eupolybothrus_fa	*Eupolybothrus_cavernicolus*
Hymenoptera3	*Fopius_arisanus*
Hymenoptera4	*Fopius_arisanus*
Hymenoptera1	*Fopius_arisanus*

Solifugae3	*Galeodes_sp*
AEG78684_green_opsin_LWb_Gryll	*Gryllus_bimaculatus*
AEG78683_green_opsin_LWa_Gryll	*Gryllus_bimaculatus*
XP_556823_AGAP006126_PB_GPROP8	*Gryllus_bimaculatus*
AEG78686_UV_opsin_Gryllus_bima	*Gryllus_bimaculatus*
AEG78685_blue_opsin_Gryllus_bi	*Gryllus_bimaculatus*
Embioptera1	*Haploembia_palaui*
Embioptera2	*Haploembia_palaui*
BAG14331_kumopsin2_Hasarius_ad	*Hasarius_adansoni*
BAG14330_kumopsin1_Hasarius_ad	*Hasarius_adansoni*
BAG14332_kumopsin3_Hasarius_ad	*Hasarius_adansoni*
BAA09132_opsin_BcRh1_Hemigraps	*Hemigrapsus_sanguinensis*
BAA09133_opsin_BcRh2_Hemigraps	*Hemigrapsus_sanguinensis*
ABI48875_opsin_c1_Holmesimysis	*Holmesimysis_costata*
AAT01077_rhodopsin_Homalodisca	*Homalodisca_vitripennis*
ABI48884_opsin_Homarus_gammaru	*Homarus_gammarus*
Hypsibius_dujardini_Hd_r_opsin	*Hypsibius_dujardini*
XP_002408319_rod_opsin_Ixodes_	*Ixodes_scapularis*
AAA02498_opsin1_lateral_Limulu	*Limulus_polyphemus*
AAA02499_opsin2_ocellar_Limulu	*Limulus_polyphemus*
ACO05013_opsin5_Limulus_polyph	*Limulus_polyphemus*
Limulus2	*Limulus_polyphemus*
Limulus1	*Limulus_polyphemus*
Psocoptera1	*Liposcelis_entomophila*
Psocoptera2	*Liposcelis_entomophila*
ABH00987_rhodopsin_Litopenaeus	*Litopenaeus_vannamei*
BAH56227_LW_opsin_Luciola_cruc	*Luciola_cruciata*
BAH56228_UV_opsin_Luciola_cruc	*Luciola_cruciata*
AAG17119_LW_opsin_Megoura_vici	*Megoura_viciae*
AAG17120_UV_A_opsin_Megoura_vi	*Megoura_viciae*
Archaeognath1	*Meinertellus_cundinamarcensis*
MeioTranscriptc76562_g1_i1_g_5	*Meiopriapulus_fijiensis*
Meio5112684_1466_10668	*Meiopriapulus_fijiensis*
Phthiraptera4	*Menopon_gallinae*
ABI48885_opsin_Mysis_diluviana	*Mysis_diluviana*
ABG37007_opsin_Rh1_Neogonodact	*Neogonodactylus_oerstedii*
ABG37009_opsin_Rh3_Neogonodact	*Neogonodactylus_oerstedii*
ABG37008_opsin_Rh2_Neogonodact	*Neogonodactylus_oerstedii*
ABI48886_opsin_c1_Neomysis_ame	*Neomysis_Armericana*
AAU95194_rhodopsin_Oncometopia	*Oncometopia_nigricans*
Oniscidea1	*Oniscidea_sp*
AFM43712_onychopsin_Ooperipatu	*Ooperipatus_hispidus*
Opilione2	*Opiliones_sp*
AAD29575_Rh3_LW_Papilio_glaucu	*Papilio_glaucus*
AAD34220_Rh1_LW_Papilio_glaucu	*Papilio_glaucus*
AAD34221_Rh2_LW_Papilio_glaucu	*Papilio_glaucus*

AAD34224_Rh4_LW_Papilio_glaucu	*Papilio_glaucus*
AAD34222_Rh5_UV_Papilio_glaucu	*Papilio_glaucus*
AAD34223_Rh6_blue_Papilio_glau	*Papilio_glaucus*
Paragordiuscomp51652_c0_seq13	*Paragordius_robustus*
Paragordiuscomp53102_c0_seq2	*Paragordius_robustus*
Paragordiuscomp54896_c0_seq1	*Paragordius_robustus*
Paragordius4	*Paragordius_robustus*
ParamacrobiotusS12_7_jcf718000	*Paramacrobiotus_richtersi*
XP_002432663_GPRop3_UNOP_Pedic	*Pediculus_humanus*
XP_002422743_GPRop2_UV_opsin_P	*Pediculus_humanus*
Blattodea1	*Periplaneta_americana*
AFM43710_onychopsin_Phalloceph	*Phallocephale_tallagandensis*
BAD66860_PrL_opsin_Pieris_rapa	*Pieris_rapae*
XP_002427337_GPRop1_LW_opsin_P	*Pieris_rapae*
BAE19944_PrUV_opsin_Pieris_rap	*Pieris_rapae*
BAE19946_PrB_opsin_Pieris_rapa	*Pieris_rapae*
BAE19945_PrV_opsin_Pieris_rapa	*Pieris_rapae*
BAG14334_kumopsin2_Plexippus_p	*Plexippus_paykulli*
BAG14333_kumopsin1_Plexippus_p	*Plexippus_paykulli*
BAG14335_kumopsin3_Plexippus_p	*Plexippus_paykulli*
AAB25036_opsin_Procambarus_cla	*Procambarus_clarkii*
Prostemmiulus_2	*Prostemmiulus_sp*
UV7_rhoPro_Rh7_like_Rhodnius_p	*Rhodnius_prolixus*
LWS_rhoPro_LW_opsin_Rhodnius_p	*Rhodnius_prolixus*
UV5_rhoPro_UV_opsin_Rhodnius_p	*Rhodnius_prolixus*
CAA56377_lop1_LW_opsin_Schisto	*Schistocerca_gregaria*
CAA56378_lop2_UV_opsin_Schisto	*Schistocerca_gregaria*
Scutigera_Scutigera_fasc62535_	*Scutigera_coleoptrata*
Strepsiptera1	*Stylops_melittae*
tetur07g05150_UV_Tetranychus_u	*Tetranychus_urticae*
tetur24g02280_UV_Tetranychus_u	*Tetranychus_urticae*
tetur12g04340_LW_Tetranychus_u	*Tetranychus_urticae*
ACH56536_LW_opsin_Thermonectus	*Thermonectus_marmoratus*
ACH56537_UV_I_opsin_Thermonect	*Thermonectus_marmoratus*
ACH56538_UV_II_opsin_Thermonec	*Thermonectus_marmoratus*
EFA03667_rhodopsin_green_TC013	*Tribolium_castaneum*
XP_970344_uv_opsin_Tribolium_c	*Tribolium_castaneum*
BAG80978_RhC_Triops_granarius	*Triops_granarius*
BAG80977_RhB_Triops_granarius	*Triops_granarius*
BAG80980_RhE_Triops_granarius	*Triops_granarius*
BAG80979_RhD_Triops_granarius	*Triops_granarius*
BAG80976_RhA_Triops_granarius	*Triops_granarius*
ACT31580_opsin_1_Uca_vomeris	*Uca_vomeris*
ACT31581_opsin_2_Uca_vomeris	*Uca_vomeris*
AAL59879_AF385333_1_LW_opsin_V	*Vanessa_cardui*
AAP49025_AF414074_1_UV_opsin_V	*Vanessa_cardui*

AAP49026_AF414075_1_blue_opsin *Vanessa_cardui*

Supplemental methods:

Genome and transcriptome assembly:

Transcriptomes originally sourced from NCBI (those from Hexapoda and Myriapoda), and new transcriptomes from Chelicerata and Priapulida were assembled in Trinity under default conditions [S1].

The assembly process of the transcriptome of *Paragordius varius* used in this study is described in detail in [S2]. The assembly process of the new tardigrade genomes followed the protocol outlined in [S3]. The assembly process of the new ecdysozoan transcriptomes in this studied followed the protocol outlined in [S4]

R-Op: The rhabdomeric opsin model

This dataset was used to construct a rhabdomeric opsin-specific matrix in PAML [37] as per Abascal et al (2008) [36]. In order to accomplish this, a phylogenetic tree was constructed under GTR+G in Phylobayes MPI 1.7 [34]. This tree was then utilised as a starting point, alongside the opsin dataset, to generate a rhabdomeric opsin specific matrix using the codeml function of PAML 4.8 [37]. The resultant matrix was then compared to the GTR matrices approximated on the same dataset by Phylobayes [34], and JTT and WAG using the cross-validation function available in Phylobayes. The r-opsin PAML-derived model proved to be superior at approximating the compositional heterogeneity of the sequences in the dataset and at anticipating saturation in the data.

This new model may prove useful for future analyses of the rhabdomeric opsins. As the amount of known data builds, these smaller functional protein family empirical models may be useful at approximating a better understanding of the group than GTR models, as they rely on a basic foundation of informative phylogenetic knowledge.

Table 3: Calibrations used within the opsin molecular clock analysis.

Divergence	Opsin sequence 1	Opsin Sequence2	MAX	MIN
Priapulida	Meio5112684_1466_10668	Paragordius4	581	519
Nematomorpha	Paragordius4	Hypsibius_dujardini_Hd_r_opsin	581	502
Tardigrada	Hypsibius_dujardini_Hd_r_opsin	AFM75824_onychopsin_Euperipato	581	524
Eutardigrada/Heterotardigrada	Hypsibius_dujardini_Hd_r_opsin	Batillipes1_scf7180001269268_N	502	72
Arthropod MWS/Arthropod UV	CCO61974_rhodopsin_2_Cupienniu	EFX81332_UV_wavelength_opsin_D	581	521
Chelicerate UV/ Insect UV	CCO61975_rhodopsin_3_Cupienniu	AAA28854_Rh3_Drosophila_melano	581	521
Crustacean UV/Insect UV	EFX81332_UV_wavelength_opsin_D	Psocoptera2	543	510
Crustacean Blue/ Insect Blue	BAG80984_opsin_RhA_Branchinell	Hymenoptera1	543	510
Chelicerate Rh7/ Pancrustacea Rh7	tetur07g05150_UV_Tetranychus_u	AAF49949_Rh7_Drosophila_melano	581	521
Crustacean Rh7/Insect Rh7	EFX70801_UNOP1_DAPPUDRAFT_3469	UV7_rhoPro_Rh7_like_Rhodnius_p	543	510
Chelicerate LWS/Mandibulate LWS	Solifugae3	Cleidogona_2	581	521
Pancrustacea MWS/Chelicerate MWS	EFX77537_LOPB1_LOPB_clade_1_15	ACO05013_opsin5_Limulus_polyph	581	521
Crustacean LWS/Insect LWS	ABI48885_opsin_Mysis_diluviana	Ephemeroptera4	581	510
Chelicerate LWS/ Pancrustacea LWS	AAA02498_opsin1_lateral_Limulu	ABI48885_opsin_Mysis_diluviana	581	521

Calibrations as per Rota Stabelli et al (2013), with the exception of Nematomorpha, which is calibrated utilizing the Nematoda calibration in Rota-Stabelli et al (2013) and Eutardigrada/Heterotardigrada, which is calibrated based on the existence of *Beorn leggi*, described as a crown eutardigrade [S5].

Table 4: Calibrations used within the species divergence tree clock analysis, based on the occurrence of the species within the fossil record, and calibrations provided by [27]. In cases where no calibration is provided by [27] directly, the age of the unit where the taxon occurs, as determined by [27], is utilised.

Taxon	Min	Max
Acinocricus_stichus	505	513
Actinarctus_doryphorus_Heterotardigrada	0	0
Aegirocassis_benmoulae	476.3	482.2
Alalcomenaeus_sp	504.5	521
Anomalocaris_canadensis	504.5	509
Antennacanthopodia_gracilis	514	521
Aysheaia_pendunculata	504.5	509
Batillipes_pennaki_Heterotardigrada	0	0
Cardiodictyon_catenulum	514	521
Chengjiangocaris_kunmingensis	514	521
Collins_monster_Burgess_Shale	504.5	509
Collins_monster_Emu_Bay	509	514
Collinsium_ciliosum	514	521
Cricocosmia_jinningensis	514	521
Diania_cactiformis	514	521
Echiniscus_testudo_Heterotardigrada	0	0
Epiperipatus_biolleyi_Peripatidae	0	0
Euperipatoides_Peripatopsidae	0	0
Fuxianhuia_xiaoshibaensis	514	521
Hadranax_augustus	514	519.5
Hallucigenia_fortis	514	521
Hallucigenia_hongmeia	514	521
Hallucigenia_sparsa	514	521
Hurdia_victoria	504.5	509

**Hypsibius_dujardini_Eutardigrada**	0	0
**Jianshanopodia_decora**	514	521
**Kerygmachela_kierkegaardi**	514	519.5
**Kuamaia_lata**	514	521
**Leanchoilia_superlata**	504.5	509
**Limulus_polyphemus**	0	0
**Luolishania_longicruris**	514	521
**Lyrarapax_unguispinus**	514	521
**Macrobiotus_cf_harmsworthi_Eutardigrada**	0	0
**Megadictyon_haikouensis**	514	521
**Metaperipatus_blainvillei_Peripatopsidae**	0	0
**Microdictyon_sinicum**	514	521
**Misszhouia_longicaudata**	514	521
**Onychodictyon_ferox**	514	521
**Onychodictyon_gracilis**	514	521
**Opabinia_regalis**	504.5	509
**Orstenotubulus_evamuellerae**	497	500.5
**Pambdelurion_whittingtoni**	514	519.5
**Paucipodia_inermis**	514	521
**Peytoia_nathorsti**	504.5	509
**Schinderhannes_bartlesi**	407	408.4
**Siberian_Orsten_tardigrade**	497	500.5
**Tertiapatus_dominicanus**	15	45
**Triops_cancriformis**	0	0
**Tubiluchus_troglodytes_Priapulida**	0	0
**Xenusion_auerswaldae**	509	514

Description of *Pambdelurion whittingtoni*

Pambdelurion whittingtoni [11] is an early Cambrian gilled lobopod from Sirius Passet, North Greenland. New material collected from the outcrop reveal details primarily preserved as reflective films. MGUH 30506 (Natural History Museum of Denmark, Geological Museum) preserves paired reflective patches at the base of the frontal appendages. These conform in preservation and position to eyes, homologous to onychophoran ocelli and tardigrade eyespots.

Supplemental Results & Discussion

New fossil data from *Pambdelurion whittingtoni*

We recovered and examined a new fossil of *Pambdelurion whittingtoni* [11] that exhibits a pair of large, sessile eyes, similar to those described recently in *Kerygmachela* [62]. Reflective pads with some relief are present at the base of each frontal appendage (Supplemental Figure 8). *Pambdelurion whittingtoni* is an early Cambrian gilled lobopod – a stem group arthropod [41] - from Sirius Passet, North Greenland [11]. This is the first recorded fossil evidence of eyes in *Pambdelurion whittingtoni*, which has previously been described primarily from its ventral surface [11]. Although distinct ommatidia cannot be discerned, we interpret these as compound eyes, due to their large size and position, corresponding to stalked eyes in the radiodontans – a closely related group of Cambrian stem arthropods that are present in fossil beds of similar age [43]. Isolated ocelli would attain much smaller sizes, and their morphology does not resemble fossil camera eyes in overall anatomy, as in *Kerygmachela* [62]. The lack of preserved ommatidia is common in Burgess Shale-type Lagerstätten preservation, such as in the Burgess Shale and Chengjiang. Emu Bay Shale contains the only record of radiodontan ommatidia [42, 43], while they are preserved as reflective patches in *Opabinia* and radiodontans from the Burgess Shale [44].

Morphological data are consistent with a gradual evolution of the compound eye in the arthropod stem lineage, from a single ocellus, to a cluster [14], followed by a sessile compound eye in this gilled lobopod grade and through to a stalked compound eye in radiodontans.

Additional References:

S1. Grabherr MG, Haas BJ, Yassour M, Levin JZ, Thompson DA, Amit I, Adiconis X, Fan L, Raychowdhury R, Zeng Q, Chen Z. 2011 Full-length transcriptome assembly from RNA-Seq data without a reference genome. *Nature biotechnology* **29**, 644.

S2. Sarkies P, Selkirk ME, Jones JT, Blok V, Boothby T, Goldstein B, Hanelt B, Ardila-Garcia A, Fast NM, Schiffer PM, Kraus C. 2015 Ancient and novel small RNA pathways compensate for the loss of piRNAs in multiple independent nematode lineages. *PLoS biology* **13**, 1002061.

S3. Arakawa K, Yoshida Y, Tomita M. 2016 Genome sequencing of a single tardigrade *Hypsibius dujardini* individual. *Scientific data* **3** 160063.

S4. Leite DJ., Baudouin-Gonzalez L., Iwasaki-Yokozawa S., Lozano-Fernandez J., Turetzek N., Akiyama-Oda Y., Prpic NM., Pisani D., Oda H., Sharma PP., McGregor AP. 2018 Homeobox Gene Duplication and Divergence in Arachnids. *MBE* **35**, 2240-2253.

S5. Cooper KW. 1964 The first fossil tardigrade: *Beorn leggi Cooper*, from Cretaceous amber. *Psyche* **71** 41-48.
