# Supplementary material for: Molecular palaeontology illuminates the evolution of ecdysozoan vision
Source: Proc Biol Sci. 2018 Dec 5;285(1892):20182180. doi: 10.1098/rspb.2018.2180 (PMC6283943; doi:10.1098/rspb.2018.2180)
Supplement: Supplemental Methods [file rspb20182180supp2.pdf]

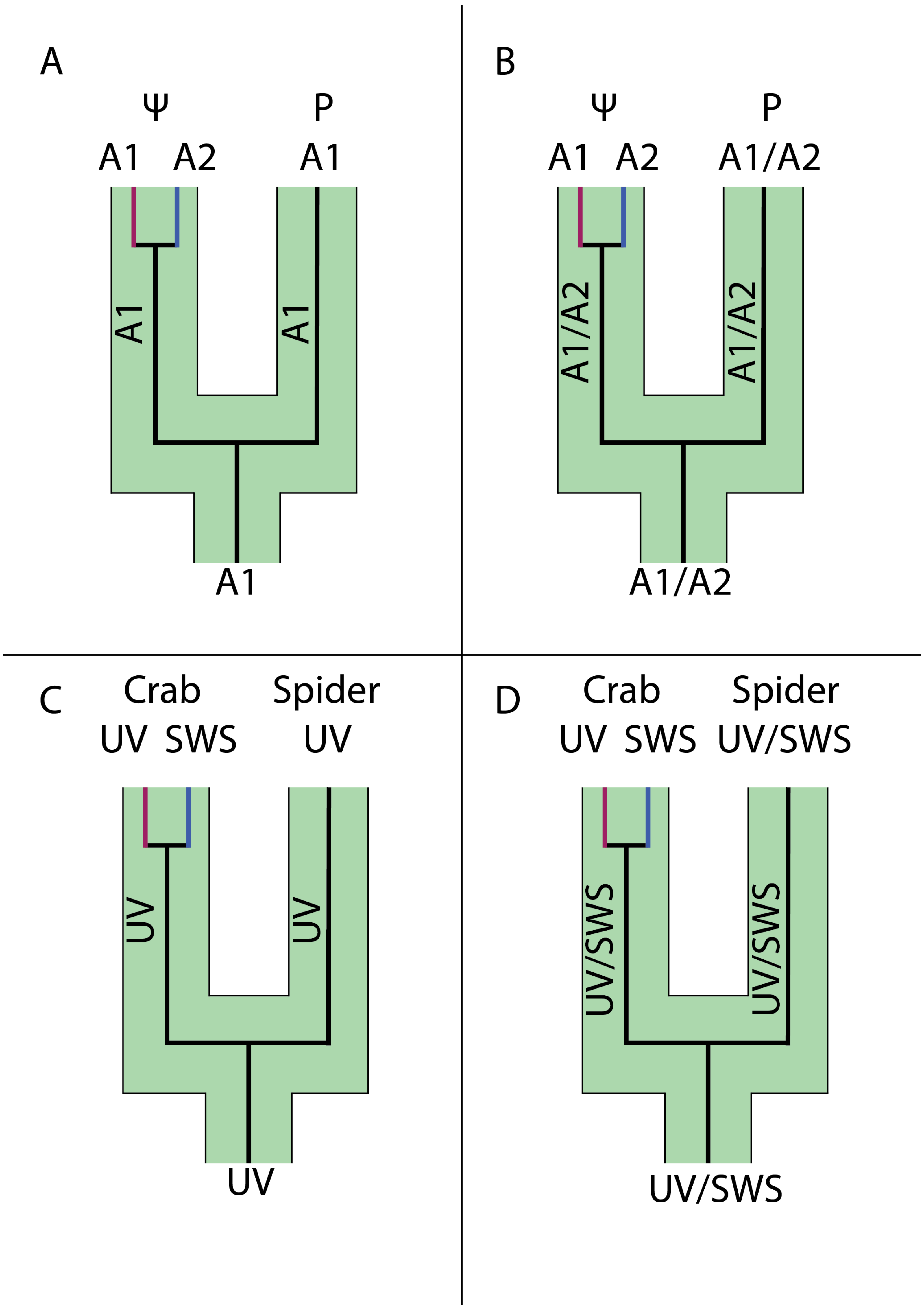
A
Ψ
A1
A2
P
A1
A1
A1
A1
B
Ψ
A1
A2
P
A1/A2
A1/A2
A1/A2
A1/A2
C
Crab
UV
SWS
Spider
UV
UV
UV
UV
D
Crab
UV
SWS
Spider
UV/SWS
UV/SWS
UV/SWS
UV/SWS

Supplemental Figure 1: A figure discussing Gene Family Nomenclature. Panels a and b display a generalised depiction of differences between the two nomenclatures, whilst figures c and d illustrate a specific example within the arthropod visual opsins. In the nomenclature utilised in this paper, the shared ancestor of the UV and SWS opsin is referred to as UV/SWS, rather than UV, even when this opsin is present in extant forms with well-characterised visual spectra, as the shared ancestral receptivity of this opsin is not necessarily within the UV.

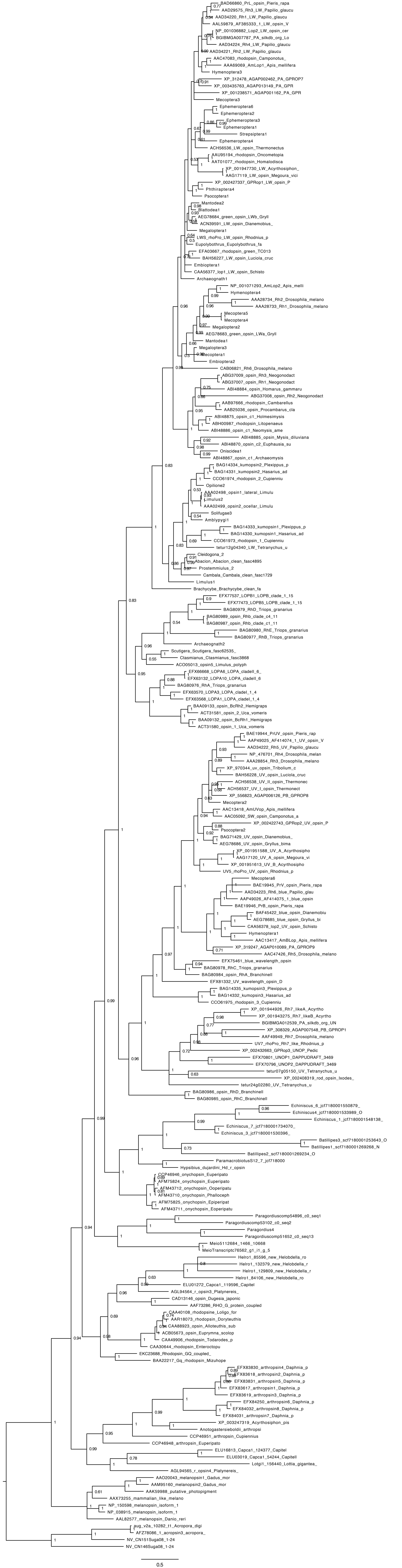
BAD66860_PrL_opsin_Pieris_rapa
AAD29575_Rh3_LW_Papilio_glaucu
AAD34220_Rh1_LW_Papilio_glaucu
AAL59879_AF385333_1_LW_opsin_V
NP_001036882_Lop2_LW_opsin_cer
BGIBMGA007787_PA_silkdb_org_Lo
AAD34224_Rh4_LW_Papilio_glaucu
AAD34221_Rh2_LW_Papilio_glaucu
AAC47083_rhodopsin_Camponotus_
AAA69069_AmLop1_Apis_mellifera
Hymenoptera3
XP_312478_AGAP002462_PA_GPROP7
XP_003435763_AGAP013149_PA_GPR
XP_001238571_AGAP001162_PA_GPR
Mecoptera3
Ephemeroptera6
Ephemeroptera2
Ephemeroptera3
Ephemeroptera1
Strepsiptera1
Ephemeroptera4
ACH56536_LW_opsin_Thermonectus
AAU95194_rhodopsin_Oncometopia
AAT01077_rhodopsin_Homalodisca
XP_001947730_LW_opsin_Acyrthosiphon_
AAG17119_LW_opsin_Megoura_vici
XP_002427337_GPRop1_LW_opsin_P
Phthiraptera4
Psocoptera1
Mantodea2
Blattodea1
AEG78684_green_opsin_LWb_Gryll
ACN39591_LW_opsin_Dianemobius_
Megaloptera1
LWS_rhoPro_LW_opsin_Rhodnius_p
Eupolybothrus_Eupolybothrus_fa
EFA03667_rhodopsin_green_TC013
BAH56227_LW_opsin_Luciola_cruc
Embioptera1
CAA56377_lop1_LW_opsin_Schisto
Archaeognath1
NP_001071293_AmLop2_Apis_melli
Hymenoptera4
AAA28734_Rh2_Drosophila_melano
AAA28733_Rh1_Drosophila_melano
Mecoptera5
Mecoptera4
Megaloptera2
AEG78683_green_opsin_LWa_Gryll
Mantodea1
Megaloptera3
Mecoptera1
Embioptera2
CAB06821_Rh6_Drosophila_melano
ABG37009_opsin_Rh3_Neogonodact
ABG37007_opsin_Rh1_Neogonodact
ABI48884_opsin_Homarus_gammaru
ABG37008_opsin_Rh2_Neogonodact
AAB97666_rhodopsin_Cambarellus
AAB25036_opsin_Procambarus_cla
ABI48875_opsin_c1_Holmesimysis
ABH00987_rhodopsin_Litopenaeus
ABI48886_opsin_c1_Neomysis_ame
ABI48885_opsin_Mysis_diluviana
ABI48870_opsin_c2_Euphausia_su
Oniscidea1
ABI48867_opsin_c1_Archaeomysis
BAG14334_kumopsin2_Plexippus_p
BAG14331_kumopsin2_Hasarius_ad
CCO61974_rhodopsin_2_Cupienniu
Opilione2
AAA02498_opsin1_lateral_Limulu
Limulus2
AAA02499_opsin2_ocellar_Limulu
Solifugae3
Amblypygi1
BAG14333_kumopsin1_Plexippus_p
BAG14330_kumopsin1_Hasarius_ad
CCO61973_rhodopsin_1_Cupienniu
tetur12g04340_LW_Tetranychus_u
Cleidogona_2
Abacion_Abacion_clean_fasc4895
Prostemmiulus_2
Cambala_Cambala_clean_fasc1729
Limulus1
Brachycybe_Brachycybe_clean_fa
EFX77537_LOPB1_LOPB_clade_1_15
EFX77473_LOPB5_LOPB_clade_1_15
BAG80979_RhD_Triops_granarius
BAG80989_opsin_Rhb_clade_c4_11
BAG80987_opsin_Rhb_clade_c1_11
BAG80980_RhE_Triops_granarius
BAG80977_RhB_Triops_granarius
Archaeognath2
Scutigera_Scutigera_fasc62535_
Ctasmianus_Ctasmianus_fasc3868
ACO05013_opsin5_Limulus_polyph
EFX66668_LOPA6_LOPA_cladeII_6_
EFX63132_LOPA10_LOPA_cladeII_6
BAG80976_RhA_Triops_granarius
EFX63570_LOPA3_LOPA_cladeI_1_4
EFX63568_LOPA1_LOPA_cladeI_1_4
BAA09133_opsin_BcRh2_Hemigraps
ACT31581_opsin_2_Uca_vomeris
BAA09132_opsin_BcRh1_Hemigraps
ACT31580_opsin_1_Uca_vomeris
BAE19944_PrUV_opsin_Pieris_rap
AAP49025_AF414074_1_UV_opsin_V
AAD34222_Rh5_UV_Papilio_glaucu
NP_476701_Rh4_Drosophila_melan
AAA28854_Rh3_Drosophila_melano
XP_970344_uv_opsin_Tribolium_c
BAH56228_UV_opsin_Luciola_cruc
ACH56538_UV_II_opsin_Thermonec
ACH56537_UV_I_opsin_Thermonect
XP_556823_AGAP006126_PB_GPROP8
Mecoptera2
AAC13418_AmUVop_Apis_mellifera
AAC05092_SW_opsin_Camponotus_a
XP_002422743_GPRop2_UV_opsin_P
Psocoptera2
BAG71429_UV_opsin_Dianemobius_
AEG78686_UV_opsin_Gryllus_bima
XP_001951588_UV_A_Acyrthosipho
AAG17120_UV_A_opsin_Megoura_vi
XP_001951613_UV_B_Acyrthosipho
UV5_rhoPro_UV_opsin_Rhodnius_p
Mecoptera6
BAE19945_PrV_opsin_Pieris_rapa
AAD34223_Rh6_blue_Papilio_glau
AAP49026_AF414075_1_blue_opsin
BAE19946_PrB_opsin_Pieris_rapa
BAF45422_blue_opsin_Dianemobiu
AEG78685_blue_opsin_Gryllus_bi
CAA56378_lop2_UV_opsin_Schisto
Hymenoptera1
AAC13417_AmBLop_Apis_mellifera
XP_319247_AGAP010089_PA_GPROP9
AAC47426_Rh5_Drosophila_melano
EFX75461_blue_wavelength_opsin
BAG80978_RhC_Triops_granarius
BAG80984_opsin_RhA_Branchinell
EFX81332_UV_wavelength_opsin_D
BAG14335_kumopsin3_Plexippus_p
BAG14332_kumopsin3_Hasarius_ad
CCO61975_rhodopsin_3_Cupienniu
XP_001944926_Rh7_likeA_Acyrtho
XP_001943275_Rh7_likeB_Acyrtho
BGIBMGA012539_PA_silkdb_org_UN
XP_308329_AGAP007548_PB_GPROP1
AAF49949_Rh7_Drosophila_melano
UV7_rhoPro_Rh7_like_Rhodnius_p
XP_002432663_GPRop3_UNOP_Pedic
EFX70801_UNOP1_DAPPUDRAFT_3469
EFX70796_UNOP2_DAPPUDRAFT_3469
tetur07g05150_UV_Tetranychus_u
XP_002408319_rod_opsin_Ixodes_
tetur24g02280_UV_Tetranychus_u
BAG80986_opsin_RhD_Branchinell
BAG80985_opsin_RhC_Branchinell
Echiniscus_6_jcf7180001550879_
Echiniscus4_jcf7180001533989_O
Echiniscus_1_jcf7180001548138_
Echiniscus_7_jcf7180001734070_
Echiniscus_3_jcf7180001530396_
Batillipes3_scf7180001253643_O
Batillipes1_scf7180001269268_N
Batillipes2_scf7180001269234_O
ParamacrobiotusS12_7_jcf718000
Hypsibius_dujardini_Hd_r_opsin
CCP46946_onychopsin_Euperipato
AFM75824_onychopsin_Epiperipat
AFM43712_onychopsin_Ooperipatu
AFM43710_onychopsin_Phalloceph
AFM75825_onychopsin_Epiperipat
AFM43711_onychopsin_Euperipatu
Paragordiuscomp54896_c0_seq1
Paragordiuscomp53102_c0_seq2
Paragordius4
Paragordiuscomp51652_c0_seq13
Meio5112684_1466_10668
MeioTranscriptc76562_g1_i1_g_5
Helro1_85596_new_Helobdella_ro
Helro1_132379_new_Helobdella_r
Helro1_129809_new_Helobdella_r
Helro1_84106_new_Helobdella_ro
ELU01272_Capca1_119596_Capitel
AGL94564_r_opsin3_Platynereis_
CAD13146_opsin_Dugesia_japonic
AAF73286_RHO_G_protein_coupled
CAA40108_rhodopsin_Loligo_for
AAR18073_rhodopsin_Doryteuthis
CAA88923_opsin_Alloteuthis_sub
ACB05673_opsin_Euprymna_scolop
CAA49906_rhodopsin_Todarodes_p
CAA30644_rhodopsin_Enteroctopu
EKC23688_Rhodopsin_GQ_coupled_
BAA22217_Gq_rhodopsin_Mizuhope
EFX83830_arthropsin4_Daphnia_p
EFX83618_arthropsin2_Daphnia_p
EFX83831_arthropsin5_Daphnia_p
EFX83617_arthropsin1_Daphnia_p
EFX83619_arthropsin3_Daphnia_p
EFX84250_arthropsin6_Daphnia_p
EFX84032_arthropsin8_Daphnia_p
EFX84031_arthropsin7_Daphnia_p
XP_003247319_Acyrthosiphon_pis
Anotogastersieboldii_arthropsi
CCP46951_arthropsin_Cupiennius
CCP46948_arthropsin_Euperipato
ELU16813_Capca1_124377_Capitel
ELU03019_Capca1_54244_Capitell
Lotgi1_156440_Lottia_gigantea_
AGL94565_r_opsin4_Platynereis_
AAO20043_melanopsin1_Gadus_mor
AAM95160_melanopsin2_Gadus_mor
AAK59988_putative_photopigment
AAX73255_mammalian_like_melano
NP_150598_melanopsin_isoform_1
NP_038915_melanopsin_isoform_1
AAL82577_melanopsin_Danio_reri
aug_v2a_10282_t1_Acropora_digi
AFZ78086_1_acropsin3_acropora_
NV_CN151Suga08_1-24
NV_CN146Suga08_1-24
0.5

Supplemental Figure 2: A preliminary tree to determine the opsin identity of the new opsins from Ecdysozoa, aligned in MUSCLE and assembled using a GTR+G model in Phylobayes.

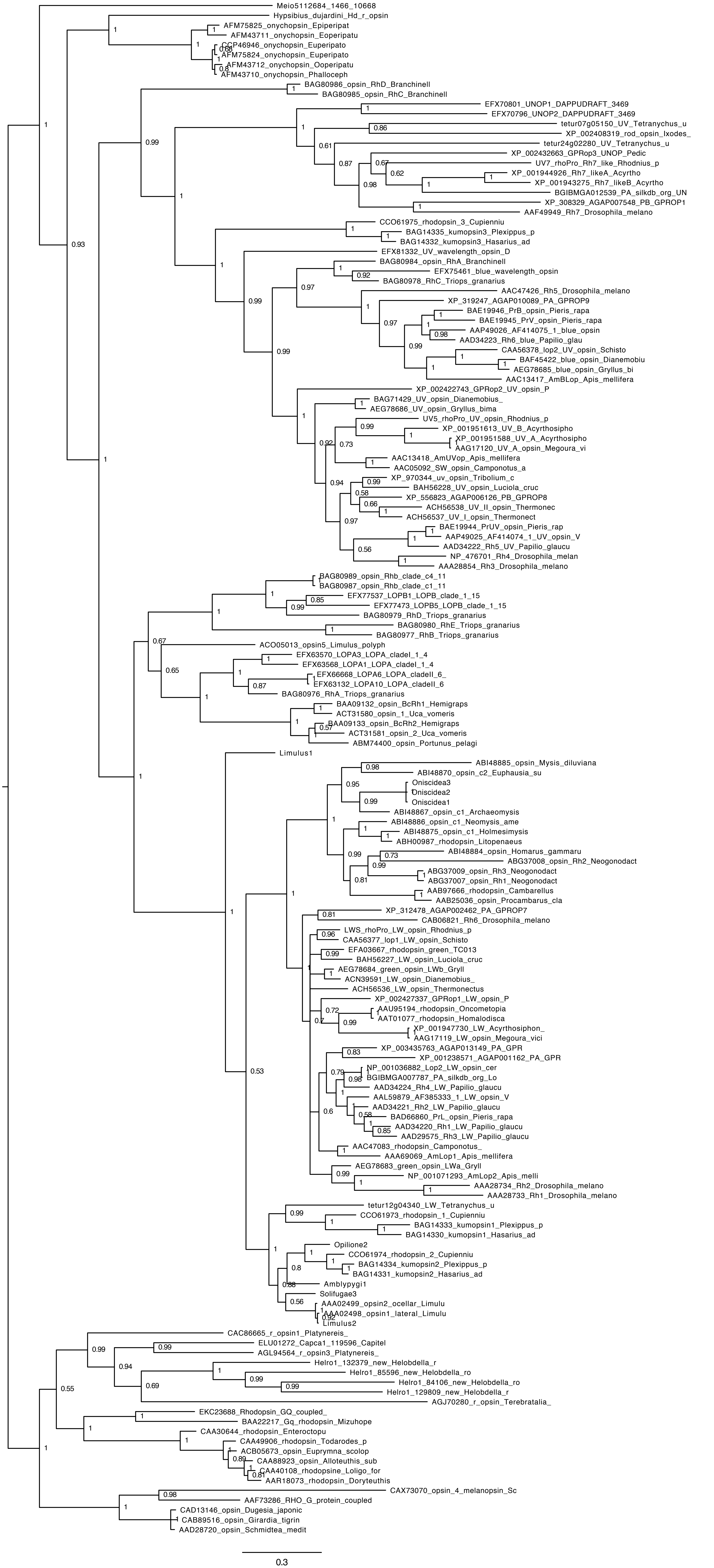
Meio5112684_1466_10668
Hypsibius_dujardini_Hd_r_opsin
AFM75825_onychopsin_Epiperipat
AFM43711_onychopsin_Eoperipatu
CCP46946_onychopsin_Euperipato
AFM75824_onychopsin_Euperipato
AFM43712_onychopsin_Ooperipatu
AFM43710_onychopsin_Phalloceph
BAG80986_opsin_RhD_Branchinell
BAG80985_opsin_RhC_Branchinell
EFX70801_UNOP1_DAPPUDRAFT_3469
EFX70796_UNOP2_DAPPUDRAFT_3469
tetur07g05150_UV_Tetranychus_u
XP_002408319_rod_opsin_Ixodes_
tetur24g02280_UV_Tetranychus_u
XP_002432663_GPRop3_UNOP_Pedic
UV7_rhoPro_Rh7_like_Rhodnius_p
XP_001944926_Rh7_likeA_Acyrtho
XP_001943275_Rh7_likeB_Acyrtho
BGIBMGA012539_PA_silkdb_org_UN
XP_308329_AGAP007548_PB_GPROP1
AAF49949_Rh7_Drosophila_melano
CCO61975_rhodopsin_3_Cupienniu
BAG14335_kumopsin3_Plexippus_p
BAG14332_kumopsin3_Hasarius_ad
EFX81332_UV_wavelength_opsin_D
BAG80984_opsin_RhA_Branchinell
EFX75461_blue_wavelength_opsin
BAG80978_RhC_Triops_granarius
AAC47426_Rh5_Drosophila_melano
XP_319247_AGAP010089_PA_GPROP9
BAE19946_PrB_opsin_Pieris_rapa
BAE19945_PrV_opsin_Pieris_rapa
AAP49026_AF414075_1_blue_opsin
AAD34223_Rh6_blue_Papilio_glau
CAA56378_lop2_UV_opsin_Schisto
BAF45422_blue_opsin_Dianemobiu
AEG78685_blue_opsin_Gryllus_bi
AAC13417_AmBLop_Apis_mellifera
XP_002422743_GPRop2_UV_opsin_P
BAG71429_UV_opsin_Dianemobius_
AEG78686_UV_opsin_Gryllus_bima
UV5_rhoPro_UV_opsin_Rhodnius_p
XP_001951613_UV_B_Acyrthosipho
XP_001951588_UV_A_Acyrthosipho
AAG17120_UV_A_opsin_Megoura_vi
AAC13418_AmUVop_Apis_mellifera
AAC05092_SW_opsin_Camponotus_a
XP_970344_uv_opsin_Tribolium_c
BAH56228_UV_opsin_Luciola_cruc
XP_556823_AGAP006126_PB_GPROP8
ACH56538_UV_II_opsin_Thermonec
ACH56537_UV_I_opsin_Thermonect
BAE19944_PrUV_opsin_Pieris_rap
AAP49025_AF414074_1_UV_opsin_V
AAD34222_Rh5_UV_Papilio_glaucu
NP_476701_Rh4_Drosophila_melan
AAA28854_Rh3_Drosophila_melano
BAG80989_opsin_Rhb_clade_c4_11
BAG80987_opsin_Rhb_clade_c1_11
EFX77537_LOPB1_LOPB_clade_1_15
EFX77473_LOPB5_LOPB_clade_1_15
BAG80979_RhD_Triops_granarius
BAG80980_RhE_Triops_granarius
BAG80977_RhB_Triops_granarius
ACO05013_opsin5_Limulus_polyph
EFX63570_LOPA3_LOPA_cladeI_1_4
EFX63568_LOPA1_LOPA_cladeI_1_4
EFX66668_LOPA6_LOPA_cladeII_6_
EFX63132_LOPA10_LOPA_cladeII_6
BAG80976_RhA_Triops_granarius
BAA09132_opsin_BcRh1_Hemigraps
ACT31580_opsin_1_Uca_vomeris
BAA09133_opsin_BcRh2_Hemigraps
ACT31581_opsin_2_Uca_vomeris
ABM74400_opsin_Portunus_pelagi
Limulus1
ABI48885_opsin_Mysis_diluviana
ABI48870_opsin_c2_Euphausia_su
Oniscidea3
Oniscidea2
Oniscidea1
ABI48867_opsin_c1_Archaeomysis
ABI48886_opsin_c1_Neomysis_ame
ABI48875_opsin_c1_Holmesimysis
ABH00987_rhodopsin_Litopenaeus
ABI48884_opsin_Homarus_gammaru
ABG37008_opsin_Rh2_Neogonodact
ABG37009_opsin_Rh3_Neogonodact
ABG37007_opsin_Rh1_Neogonodact
AAB97666_rhodopsin_Cambarellus
AAB25036_opsin_Procambarus_cla
XP_312478_AGAP002462_PA_GPROP7
CAB06821_Rh6_Drosophila_melano
LWS_rhoPro_LW_opsin_Rhodnius_p
CAA56377_lop1_LW_opsin_Schisto
EFA03667_rhodopsin_green_TC013
BAH56227_LW_opsin_Luciola_cruc
AEG78684_green_opsin_LWb_Gryll
ACN39591_LW_opsin_Dianemobius_
ACH56536_LW_opsin_Thermonectus
XP_002427337_GPRop1_LW_opsin_P
AAU95194_rhodopsin_Oncometopia
AAT01077_rhodopsin_Homalodisca
XP_001947730_LW_Acyrthosiphon_
AAG17119_LW_opsin_Megoura_vici
XP_003435763_AGAP013149_PA_GPR
XP_001238571_AGAP001162_PA_GPR
NP_001036882_Lop2_LW_opsin_cer
BGIBMGA007787_PA_silkdb_org_Lo
AAD34224_Rh4_LW_Papilio_glaucu
AAL59879_AF385333_1_LW_opsin_V
AAD34221_Rh2_LW_Papilio_glaucu
BAD66860_PrL_opsin_Pieris_rapa
AAD34220_Rh1_LW_Papilio_glaucu
AAD29575_Rh3_LW_Papilio_glaucu
AAC47083_rhodopsin_Camponotus_
AAA69069_AmLop1_Apis_mellifera
AEG78683_green_opsin_LWa_Gryll
NP_001071293_AmLop2_Apis_melli
AAA28734_Rh2_Drosophila_melano
AAA28733_Rh1_Drosophila_melano
tetur12g04340_LW_Tetranychus_u
CCO61973_rhodopsin_1_Cupienniu
BAG14333_kumopsin1_Plexippus_p
BAG14330_kumopsin1_Hasarius_ad
Opilione2
CCO61974_rhodopsin_2_Cupienniu
BAG14334_kumopsin2_Plexippus_p
BAG14331_kumopsin2_Hasarius_ad
Amblypygi1
Solifugae3
AAA02499_opsin2_ocellar_Limulu
AAA02498_opsin1_lateral_Limulu
Limulus2
CAC86665_r_opsin1_Platynereis_
ELU01272_Capca1_119596_Capitel
AGL94564_r_opsin3_Platynereis_
Helro1_132379_new_Helobdella_r
Helro1_85596_new_Helobdella_ro
Helro1_84106_new_Helobdella_ro
Helro1_129809_new_Helobdella_r
AGJ70280_r_opsin_Terebratalia_
EKC23688_Rhodopsin_GQ_coupled_
BAA22217_Gq_rhodopsin_Mizuhope
CAA30644_rhodopsin_Enteroctopu
CAA49906_rhodopsin_Todarodes_p
ACB05673_opsin_Euprymna_scolop
CAA88923_opsin_Alloteuthis_sub
CAA40108_rhodopsine_Loligo_for
AAR18073_rhodopsin_Doryteuthis
CAX73070_opsin_4_melanopsin_Sc
AAF73286_RHO_G_protein_coupled
CAD13146_opsin_Dugesia_japonic
CAB89516_opsin_Girardia_tigrin
AAD28720_opsin_Schmidtea_medit
0.3

Supplemental Figure 3: A preliminary tree to determine the opsin identity of the new opsins from Ecdysozoa, aligned in PRANK and assembled using a GTR+G model in Phylobayes.

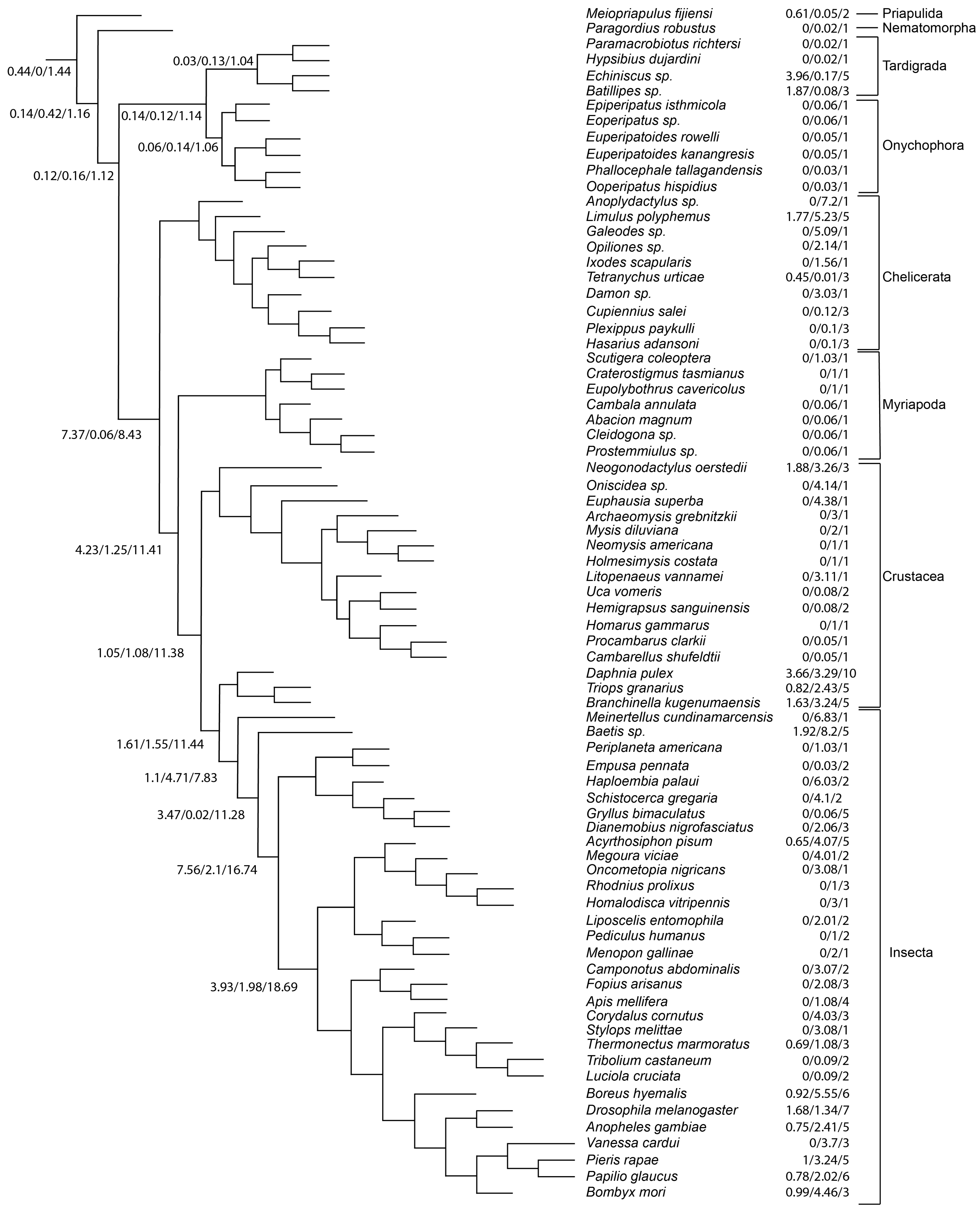

0.44/0/1.44
0.14/0.42/1.16
0.12/0.16/1.12
0.03/0.13/1.04
0.14/0.12/1.14
0.06/0.14/1.06
7.37/0.06/8.43
4.23/1.25/11.41
1.05/1.08/11.38
1.61/1.55/11.44
1.1/4.71/7.83
3.47/0.02/11.28
7.56/2.1/16.74
3.93/1.98/18.69
Meiopriapulus fijiensi 0.61/0.05/2
Paragordius robustus 0/0.02/1
Paramacrobiotus richtersi 0/0.02/1
Hypsibius dujardini 0/0.02/1
Echiniscus sp. 3.96/0.17/5
Batillipes sp. 1.87/0.08/3
Epiperipatus isthmicola 0/0.06/1
Eoperipatus sp. 0/0.06/1
Euperipatoides rowelli 0/0.05/1
Euperipatoides kanangresis 0/0.05/1
Phallocephale tallagandensis 0/0.03/1
Ooperipatus hispidius 0/0.03/1
Anoplydactylus sp. 0/7.2/1
Limulus polyphemus 1.77/5.23/5
Galeodes sp. 0/5.09/1
Opiliones sp. 0/2.14/1
Ixodes scapularis 0/1.56/1
Tetranychus urticae 0.45/0.01/3
Damon sp. 0/3.03/1
Cupiennius salei 0/0.12/3
Plexippus paykulli 0/0.1/3
Hasarius adansoni 0/0.1/3
Scutigera coleoptera 0/1.03/1
Craterostigmus tasmianus 0/1/1
Eupolybothrus cavericolus 0/1/1
Cambala annulata 0/0.06/1
Abacion magnum 0/0.06/1
Cleidogona sp. 0/0.06/1
Prostemmiulus sp. 0/0.06/1
Neogonodactylus oerstedii 1.88/3.26/3
Oniscidea sp. 0/4.14/1
Euphausia superba 0/4.38/1
Archaeomysis grebnitzkii 0/3/1
Mysis diluviana 0/2/1
Neomysis americana 0/1/1
Holmesimysis costata 0/1/1
Litopenaeus vannamei 0/3.11/1
Uca vomeris 0/0.08/2
Hemigrapsus sanguinensis 0/0.08/2
Homarus gammarus 0/1/1
Procambarus clarkii 0/0.05/1
Cambarellus shufeldtii 0/0.05/1
Daphnia pulex 3.66/3.29/10
Triops granarius 0.82/2.43/5
Branchinella kugenumaensis 1.63/3.24/5
Meinertellus cundinamarcensis 0/6.83/1
Baetis sp. 1.92/8.2/5
Periplaneta americana 0/1.03/1
Empusa pennata 0/0.03/2
Haploembia palaui 0/6.03/2
Schistocerca gregaria 0/4.1/2
Gryllus bimaculatus 0/0.06/5
Dianemobius nigrofasciatus 0/2.06/3
Acyrthosiphon pisum 0.65/4.07/5
Megoura viciae 0/4.01/2
Oncometopia nigricans 0/3.08/1
Rhodnius prolixus 0/1/3
Homalodisca vitripennis 0/3/1
Liposcelis entomophila 0/2.01/2
Pediculus humanus 0/1/2
Menopon gallinae 0/2/1
Camponotus abdominalis 0/3.07/2
Fopius arisanus 0/2.08/3
Apis mellifera 0/1.08/4
Corydalus cornutus 0/4.03/3
Stylops melittae 0/3.08/1
Thermonectus marmoratus 0.69/1.08/3
Tribolium castaneum 0/0.09/2
Luciola cruciata 0/0.09/2
Boreus hyemalis 0.92/5.55/6
Drosophila melanogaster 1.68/1.34/7
Anopheles gambiae 0.75/2.41/5
Vanessa cardui 0/3.7/3
Pieris rapae 1/3.24/5
Papilio glaucus 0.78/2.02/6
Bombyx mori 0.99/4.46/3
Priapulida
Nematomorpha
Tardigrada
Onychophora
Chelicerata
Myriapoda
Crustacea
Insecta

Supplemental Figure 4: The Gains/Losses/Duplications expressed at key nodes throughout the ecdysozoan visual opsin tree, as deduced by an ALE analysis.

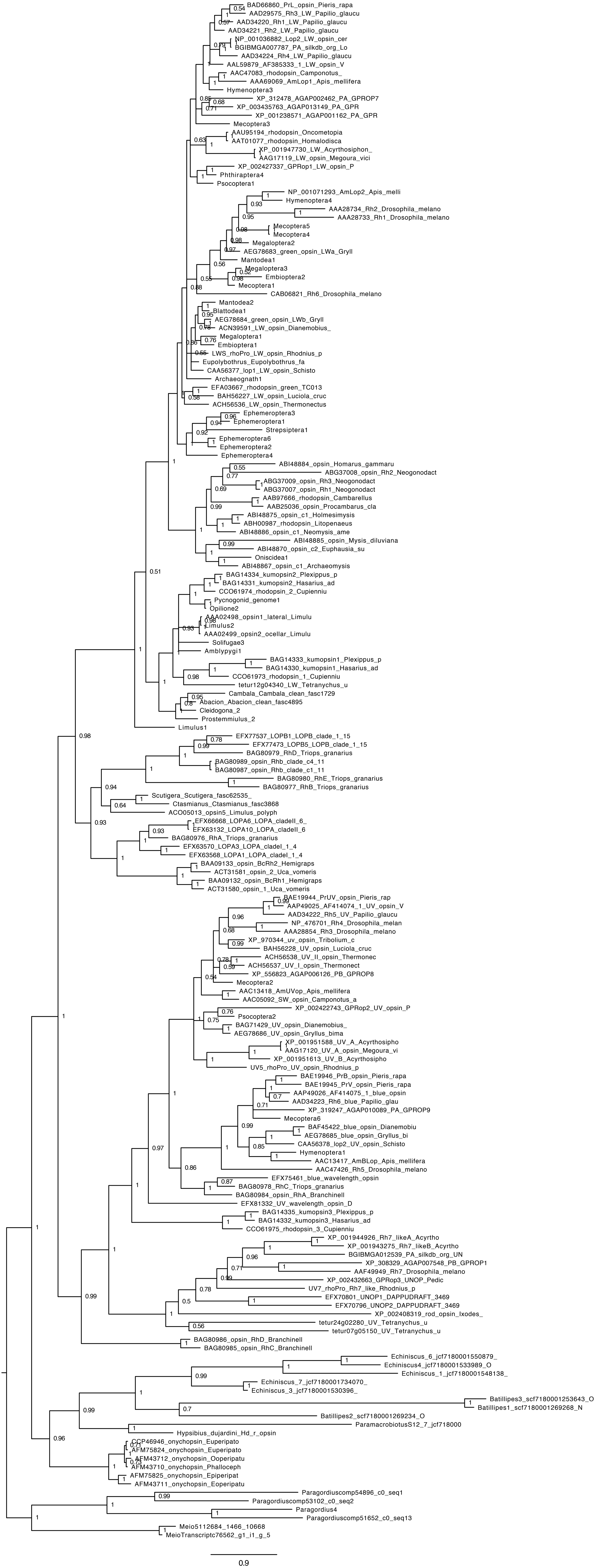
BAD66860_PrL_opsin_Pieris_rapa
AAD29575_Rh3_LW_Papilio_glaucu
AAD34220_Rh1_LW_Papilio_glaucu
AAD34221_Rh2_LW_Papilio_glaucu
NP_001036882_Lop2_LW_opsin_cer
BGIBMGA007787_PA_silkdb_org_Lo
AAD34224_Rh4_LW_Papilio_glaucu
AAL59879_AF385333_1_LW_opsin_V
AAC47083_rhodopsin_Camponotus_
AAA69069_AmLop1_Apis_mellifera
Hymenoptera3
XP_312478_AGAP002462_PA_GPROP7
XP_003435763_AGAP013149_PA_GPR
XP_001238571_AGAP001162_PA_GPR
Mecoptera3
AAU95194_rhodopsin_Oncometopia
AAT01077_rhodopsin_Homalodisca
XP_001947730_LW_Acyrthosiphon_
AAG17119_LW_opsin_Megoura_vici
XP_002427337_GPRop1_LW_opsin_P
Phthiraptera4
Psocoptera1
NP_001071293_AmLop2_Apis_melli
Hymenoptera4
AAA28734_Rh2_Drosophila_melano
AAA28733_Rh1_Drosophila_melano
Mecoptera5
Mecoptera4
Megaloptera2
AEG78683_green_opsin_LWa_Gryll
Mantodea1
Megaloptera3
Embioptera2
Mecoptera1
CAB06821_Rh6_Drosophila_melano
Mantodea2
Blattodea1
AEG78684_green_opsin_LWb_Gryll
ACN39591_LW_opsin_Dianemobius_
Megaloptera1
Embioptera1
LWS_rhoPro_LW_opsin_Rhodnius_p
Eupolybothrus_Eupolybothrus_fa
CAA56377_lop1_LW_opsin_Schisto
Archaeognath1
EFA03667_rhodopsin_green_TC013
BAH56227_LW_opsin_Luciola_cruc
ACH56536_LW_opsin_Thermonectus
Ephemeroptera3
Ephemeroptera1
Strepsiptera1
Ephemeroptera6
Ephemeroptera2
Ephemeroptera4
ABI48884_opsin_Homarus_gammaru
ABG37008_opsin_Rh2_Neogonodact
ABG37009_opsin_Rh3_Neogonodact
ABG37007_opsin_Rh1_Neogonodact
AAB97666_rhodopsin_Cambarellus
AAB25036_opsin_Procambarus_cla
ABI48875_opsin_c1_Holmesimysis
ABH00987_rhodopsin_Litopenaeus
ABI48886_opsin_c1_Neomysis_ame
ABI48885_opsin_Mysis_diluviana
ABI48870_opsin_c2_Euphausia_su
Oniscidea1
ABI48867_opsin_c1_Archaeomysis
BAG14334_kumopsin2_Plexippus_p
BAG14331_kumopsin2_Hasarius_ad
CCO61974_rhodopsin_2_Cupienniu
Pycnogonid_genome1
Opilione2
AAA02498_opsin1_lateral_Limulu
Limulus2
AAA02499_opsin2_ocellar_Limulu
Solifugae3
Amblypygi1
BAG14333_kumopsin1_Plexippus_p
BAG14330_kumopsin1_Hasarius_ad
CCO61973_rhodopsin_1_Cupienniu
tetur12g04340_LW_Tetranychus_u
Cambala_Cambala_clean_fasc1729
Abacion_Abacion_clean_fasc4895
Cleidogona_2
Prostemmiulus_2
Limulus1
EFX77537_LOPB1_LOPB_clade_1_15
EFX77473_LOPB5_LOPB_clade_1_15
BAG80979_RhD_Triops_granarius
BAG80989_opsin_Rhb_clade_c4_11
BAG80987_opsin_Rhb_clade_c1_11
BAG80980_RhE_Triops_granarius
BAG80977_RhB_Triops_granarius
Scutigera_Scutigera_fasc62535_
Ctasmianus_Ctasmianus_fasc3868
ACO05013_opsin5_Limulus_polyph
EFX66668_LOPA6_LOPA_cladeII_6_
EFX63132_LOPA10_LOPA_cladeII_6
BAG80976_RhA_Triops_granarius
EFX63570_LOPA3_LOPA_cladeI_1_4
EFX63568_LOPA1_LOPA_cladeI_1_4
BAA09133_opsin_BcRh2_Hemigraps
ACT31581_opsin_2_Uca_vomeris
BAA09132_opsin_BcRh1_Hemigraps
ACT31580_opsin_1_Uca_vomeris
BAE19944_PrUV_opsin_Pieris_rap
AAP49025_AF414074_1_UV_opsin_V
AAD34222_Rh5_UV_Papilio_glaucu
NP_476701_Rh4_Drosophila_melan
AAA28854_Rh3_Drosophila_melano
XP_970344_uv_opsin_Tribolium_c
BAH56228_UV_opsin_Luciola_cruc
ACH56538_UV_II_opsin_Thermonec
ACH56537_UV_I_opsin_Thermonect
XP_556823_AGAP006126_PB_GPROP8
Mecoptera2
AAC13418_AmUVop_Apis_mellifera
AAC05092_SW_opsin_Camponotus_a
XP_002422743_GPRop2_UV_opsin_P
Psocoptera2
BAG71429_UV_opsin_Dianemobius_
AEG78686_UV_opsin_Gryllus_bima
XP_001951588_UV_A_Acyrthosipho
AAG17120_UV_A_opsin_Megoura_vi
XP_001951613_UV_B_Acyrthosipho
UV5_rhoPro_UV_opsin_Rhodnius_p
BAE19946_PrB_opsin_Pieris_rapa
BAE19945_PrV_opsin_Pieris_rapa
AAP49026_AF414075_1_blue_opsin
AAD34223_Rh6_blue_Papilio_glau
XP_319247_AGAP010089_PA_GPROP9
Mecoptera6
BAF45422_blue_opsin_Dianemobiu
AEG78685_blue_opsin_Gryllus_bi
CAA56378_lop2_UV_opsin_Schisto
Hymenoptera1
AAC13417_AmBLop_Apis_mellifera
AAC47426_Rh5_Drosophila_melano
EFX75461_blue_wavelength_opsin
BAG80978_RhC_Triops_granarius
BAG80984_opsin_RhA_Branchinell
EFX81332_UV_wavelength_opsin_D
BAG14335_kumopsin3_Plexippus_p
BAG14332_kumopsin3_Hasarius_ad
CCO61975_rhodopsin_3_Cupienniu
XP_001944926_Rh7_likeA_Acyrtho
XP_001943275_Rh7_likeB_Acyrtho
BGIBMGA012539_PA_silkdb_org_UN
XP_308329_AGAP007548_PB_GPROP1
AAF49949_Rh7_Drosophila_melano
XP_002432663_GPRop3_UNOP_Pedic
UV7_rhoPro_Rh7_like_Rhodnius_p
EFX70801_UNOP1_DAPPUDRAFT_3469
EFX70796_UNOP2_DAPPUDRAFT_3469
XP_002408319_rod_opsin_Ixodes_
tetur24g02280_UV_Tetranychus_u
tetur07g05150_UV_Tetranychus_u
BAG80986_opsin_RhD_Branchinell
BAG80985_opsin_RhC_Branchinell
Echiniscus_6_jcf7180001550879_
Echiniscus4_jcf7180001533989_O
Echiniscus_1_jcf7180001548138_
Echiniscus_7_jcf7180001734070_
Echiniscus_3_jcf7180001530396_
Batillipes3_scf7180001253643_O
Batillipes1_scf7180001269268_N
Batillipes2_scf7180001269234_O
ParamacrobiotusS12_7_jcf718000
Hypsibius_dujardini_Hd_r_opsin
CCP46946_onychopsin_Euperipato
AFM75824_onychopsin_Euperipato
AFM43712_onychopsin_Ooperipatu
AFM43710_onychopsin_Phalloceph
AFM75825_onychopsin_Epiperipat
AFM43711_onychopsin_Ooperipatu
Paragordiuscomp54896_c0_seq1
Paragordiuscomp53102_c0_seq2
Paragordius4
Paragordiuscomp51652_c0_seq13
Meio5112684_1466_10668
MeioTranscriptc76562_g1_i1_g_5
0.9

Supplemental Figure 5: The ecdysozoan visual opsin tree aligned in MUSCLE and assembled under the GTR+G model in Phylobayes. Numbers expressed at each node are posterior probability values.

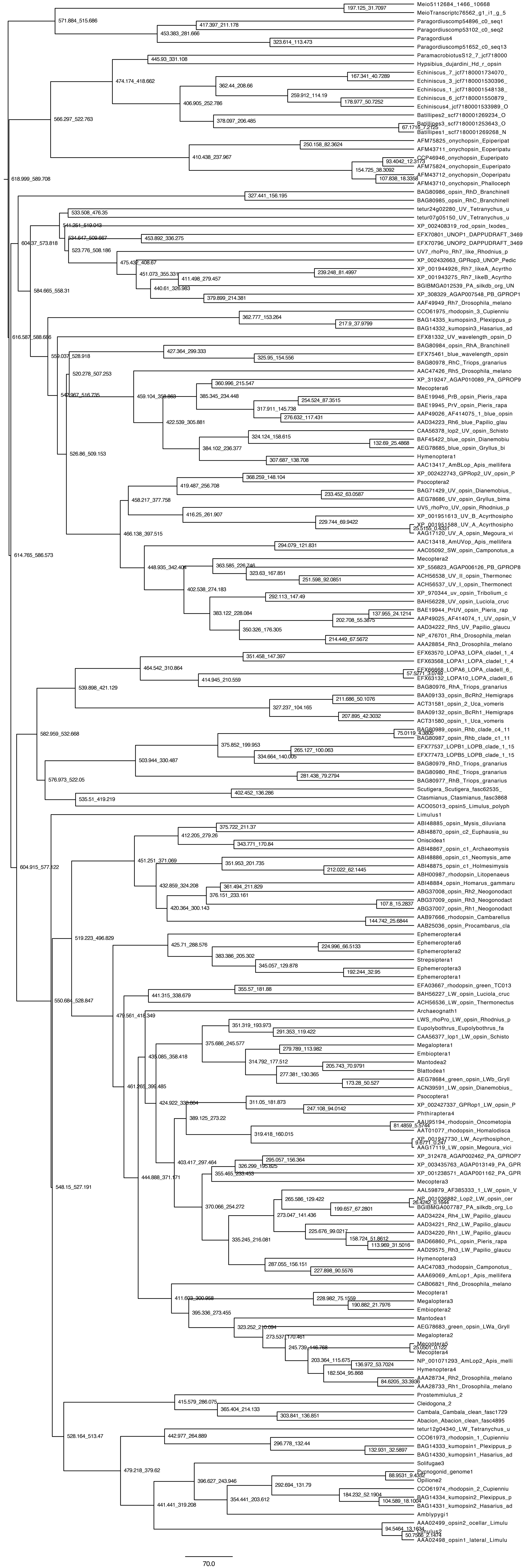

Meio5112684_1466_10668
197.125_31.7097
MeioTranscriptc76562_g1_i1_g_5
571.884_515.686
Paragordiuscomp54896_c0_seq1
417.397_211.178
Paragordiuscomp53102_c0_seq2
453.383_281.666
Paragordius4
323.614_113.473
Paragordiuscomp51652_c0_seq13
ParamacrobiotusS12_7_jcf718000
445.93_331.108
Hypsibius_dujardini_Hd_r_opsin
Echiniscus_7_jcf7180001734070_
167.341_40.7289
474.174_418.662
Echiniscus_3_jcf7180001530396_
362.44_208.66
Echiniscus_1_jcf7180001548138_
259.912_114.19
Echiniscus_6_jcf7180001550879_
178.977_50.7252
406.905_252.786
Echiniscus4_jcf7180001533989_O
566.297_522.763
Batillipes2_scf7180001269234_O
378.097_206.485
Batillipes3_scf7180001253643_O
67.1715_7.2125
Batillipes1_scf7180001269268_N
AFM75825_onychopsin_Epiperipat
250.158_82.3624
AFM43711_onychopsin_Eoperipatu
410.438_237.967
CCP46946_onychopsin_Euperipato
93.4042_12.3173
AFM75824_onychopsin_Euperipato
154.725_38.3092
AFM43712_onychopsin_Ooperipatu
107.838_18.3358
AFM43710_onychopsin_Phalloceph
618.999_589.708
BAG80986_opsin_RhD_Branchinell
327.441_156.195
BAG80985_opsin_RhC_Branchinell
533.508_476.35
tetur24g02280_UV_Tetranychus_u
tetur07g05150_UV_Tetranychus_u
541.251_519.043
XP_002408319_rod_opsin_Ixodes_
604.37_573.818
534.647_509.667
EFX70801_UNOP1_DAPPUDRAFT_3469
453.892_336.275
EFX70796_UNOP2_DAPPUDRAFT_3469
523.776_508.186
UV7_rhoPro_Rh7_like_Rhodnius_p
475.432_408.67
XP_002432663_GPRop3_UNOP_Pedic
XP_001944926_Rh7_likeA_Acyrtho
239.248_81.4997
451.073_355.331
411.498_279.457
XP_001943275_Rh7_likeB_Acyrtho
BGIBMGA012539_PA_silkdb_org_UN
440.61_326.983
XP_308329_AGAP007548_PB_GPROP1
379.899_214.381
AAF49949_Rh7_Drosophila_melano
584.665_558.31
CCO61975_rhodopsin_3_Cupienniu
362.777_153.264
BAG14335_kumopsin3_Plexippus_p
217.9_37.9799
BAG14332_kumopsin3_Hasarius_ad
616.587_588.686
EFX81332_UV_wavelength_opsin_D
BAG80984_opsin_RhA_Branchinell
427.364_299.333
559.037_528.918
EFX75461_blue_wavelength_opsin
325.95_154.556
BAG80978_RhC_Triops_granarius
520.278_507.253
AAC47426_Rh5_Drosophila_melano
XP_319247_AGAP010089_PA_GPROP9
360.996_215.547
542.967_516.735
459.104_358.863
Mecopetera6
385.345_234.448
BAE19946_PrB_opsin_Pieris_rapa
254.524_87.3515
BAE19945_PrV_opsin_Pieris_rapa
317.911_145.738
AAP49026_AF414075_1_blue_opsin
276.632_117.431
AAD34223_Rh6_blue_Papilio_glau
422.539_305.881
CAA56378_lop2_UV_opsin_Schisto
324.124_158.615
BAF45422_blue_opsin_Dianemobiu
132.69_25.4868
384.102_236.377
AEG78685_blue_opsin_Gryllus_bi
526.86_509.153
Hymenoptera1
307.687_138.708
AAC13417_AmBLop_Apis_mellifera
XP_002422743_GPRop2_UV_opsin_P
368.259_148.104
419.487_256.708
Psocoptera2
BAG71429_UV_opsin_Dianemobius_
233.452_63.0587
458.217_377.758
AEG78686_UV_opsin_Gryllus_bima
UV5_rhoPro_UV_opsin_Rhodnius_p
416.25_261.907
XP_001951613_UV_B_Acyrthosipho
229.744_69.9422
466.138_397.515
XP_001951588_UV_A_Acyrthosipho
25.5155_0.4331
AAG17120_UV_A_opsin_Megoura_vi
614.765_586.573
AAC13418_AmUVop_Apis_mellifera
294.079_121.831
AAC05092_SW_opsin_Camponotus_a
448.935_342.404
Mecopetera2
363.585_226.746
XP_556823_AGAP006126_PB_GPROP8
323.63_167.851
ACH56538_UV_II_opsin_Thermonec
251.598_92.0851
402.538_274.183
ACH56537_UV_I_opsin_Thermonect
XP_970344_uv_opsin_Tribolium_c
292.113_147.49
BAH56228_UV_opsin_Luciola_cruc
383.122_228.084
BAE19944_PrUV_opsin_Pieris_rap
137.955_24.1214
202.708_55.3875
AAP49025_AF414074_1_UV_opsin_V
350.326_176.305
AAD34222_Rh5_UV_Papilio_glaucu
NP_476701_Rh4_Drosophila_melan
214.449_67.5672
AAA28854_Rh3_Drosophila_melano
EFX63570_LOPA3_LOPA_cladeI_1_4
351.458_147.397
464.542_310.864
EFX63568_LOPA1_LOPA_cladeI_1_4
EFX66668_LOPA6_LOPA_cladeII_6
57.5271_3.0749
414.945_210.559
EFX63132_LOPA10_LOPA_cladeII_6
539.898_421.129
BAG80976_RhA_Triops_granarius
BAA09133_opsin_BcRh2_Hemigraps
211.686_50.1076
327.237_104.165
ACT31581_opsin_2_Uca_vomeris
BAA09132_opsin_BcRh1_Hemigraps
207.895_42.3032
ACT31580_opsin_1_Uca_vomeris
582.959_532.668
BAG80989_opsin_Rhb_clade_c4_11
75.0119_4.3805
375.852_199.953
BAG80987_opsin_Rhb_clade_c1_11
EFX77537_LOPB1_LOPB_clade_1_15
265.127_100.063
503.944_330.487
334.664_140.005
EFX77473_LOPB5_LOPB_clade_1_15
BAG80979_RhD_Triops_granarius
576.973_522.05
281.438_79.2794
BAG80980_RhE_Triops_granarius
BAG80977_RhB_Triops_granarius
Scutigera_Scutigera_fasc62535_
402.452_136.286
535.51_419.219
Ctasmianus_Ctasmianus_fasc3868
ACO05013_opsin5_Limulus_polyph
Limulus1
604.915_577.122
ABI48885_opsin_Mysis_diluviana
375.722_211.37
412.205_279.26
ABI48870_opsin_c2_Euphausia_su
343.771_170.84
Oniscidea1
451.251_371.069
ABI48867_opsin_c1_Archaeomysis
ABI48886_opsin_c1_Neomysis_ame
351.953_201.735
ABI48875_opsin_c1_Holmesimysis
212.022_62.1445
432.859_324.208
ABH00987_rhodopsin_Litopenaeus
ABI48884_opsin_Homarus_gammaru
361.494_211.829
ABG37008_opsin_Rh2_Neogonodact
376.151_233.161
420.364_300.143
ABG37009_opsin_Rh3_Neogonodact
107.8_15.2837
ABG37007_opsin_Rh1_Neogonodact
AAB97666_rhodopsin_Cambarellus
144.742_25.6844
519.223_496.829
AAB25036_opsin_Procambarus_cla
Ephemeroptera4
425.71_288.576
Ephemeroptera6
224.996_66.5133
383.386_205.302
Ephemeroptera2
Strepsiptera1
345.057_129.878
Ephemeroptera3
192.244_32.95
Ephemeroptera1
EFA03667_rhodopsin_green_TC013
355.57_181.88
441.315_338.679
BAH56227_LW_opsin_Luciola_cruc
550.684_528.847
ACH56536_LW_opsin_Thermonectus
479.561_418.349
Archaeognath1
LWS_rhoPro_LW_opsin_Rhodnius_p
351.319_193.973
291.353_119.422
Eupolybothrus_Eupolybothrus_fa
375.686_245.577
CAA56377_lop1_LW_opsin_Schisto
435.085_358.418
Megaloptera1
279.789_113.982
Embioptera1
314.792_177.512
Mantodea2
205.743_70.9791
277.381_130.365
Blattodea1
AEG78684_green_opsin_LWb_Gryll
173.28_50.527
461.265_399.485
ACN39591_LW_opsin_Dianemobius_
Psocoptera1
311.05_181.873
424.922_338.804
XP_002427337_GPRop1_LW_opsin_P
247.108_94.0142
Phthiraptera4
389.125_273.22
AAU95194_rhodopsin_Oncometopia
81.4859_5.5744
319.418_160.015
AAT01077_rhodopsin_Homalodisca
XP_001947730_LW_Acyrthosiphon_
9.6771_0.247
AAG17119_LW_opsin_Megoura_vici
403.417_297.464
XP_312478_AGAP002462_PA_GPROP7
295.057_156.364
326.299_195.825
XP_003435763_AGAP013149_PA_GPR
355.465_233.453
XP_001238571_AGAP001162_PA_GPR
444.888_371.171
Mecopetera3
548.15_527.191
AAL59879_AF385333_1_LW_opsin_V
NP_001036882_Lop2_LW_opsin_cer
265.586_129.422
26.4242_0.1644
370.066_254.272
199.657_67.2801
BGIBMGA007787_PA_silkdb_org_Lo
273.047_141.436
AAD34224_Rh4_LW_Papilio_glaucu
AAD34221_Rh2_LW_Papilio_glaucu
225.676_99.0217
AAD34220_Rh1_LW_Papilio_glaucu
158.724_51.8612
335.245_216.081
BAD66860_PrL_opsin_Pieris_rapa
113.969_31.5016
AAD29575_Rh3_LW_Papilio_glaucu
Hymenoptera3
287.055_156.151
AAC47083_rhodopsin_Camponotus_
227.898_90.5576
AAA69069_AmLop1_Apis_mellifera
CAB06821_Rh6_Drosophila_melano
411.603_300.958
Mecopetera1
228.982_75.1559
Megaloptera3
190.882_21.7976
395.336_273.455
Embioptera2
Mantodea1
323.252_210.094
AEG78683_green_opsin_LWa_Gryll
273.537_170.461
Megaloptera2
Mecopetera5
245.739_146.768
25.0501_0.122
Mecopetera4
203.364_115.675
NP_001071293_AmLop2_Apis_melli
136.972_53.7024
Hymenoptera4
182.504_95.868
AAA28734_Rh2_Drosophila_melano
84.6205_33.3936
AAA28733_Rh1_Drosophila_melano
Prostemmiulus_2
415.579_286.075
Cleidogona_2
365.404_214.133
Cambala_Cambala_clean_fasc1729
303.841_136.851
Abacion_Abacion_clean_fasc4895
tetur12g04340_LW_Tetranychus_u
528.164_513.47
442.977_264.889
CCO61973_rhodopsin_1_Cupienniu
296.778_132.44
BAG14333_kumopsin1_Plexippus_p
132.931_32.5897
BAG14330_kumopsin1_Hasarius_ad
479.218_379.62
Solifugae3
Pycnogonid_genome1
88.9531_9.4352
396.627_243.946
Opilione2
292.694_131.79
CCO61974_rhodopsin_2_Cupienniu
184.232_52.1904
354.441_203.612
BAG14334_kumopsin2_Plexippus_p
104.589_18.1008
441.441_319.208
BAG14331_kumopsin2_Hasarius_ad
Amblypygi1
AAA02499_opsin2_ocellar_Limulu
94.5464_13.1634
Limulus2
50.7566_2.1474
AAA02498_opsin1_lateral_Limulu
70.0

Supplemental Figure 6: The ecdysozoan visual opsin tree aligned in MUSCLE and assembled under the GTR+G model in Phylobayes. Numbers expressed at each node are age in Ma, the result of a molecular clock analysis under the CIR model in Phylobayes.

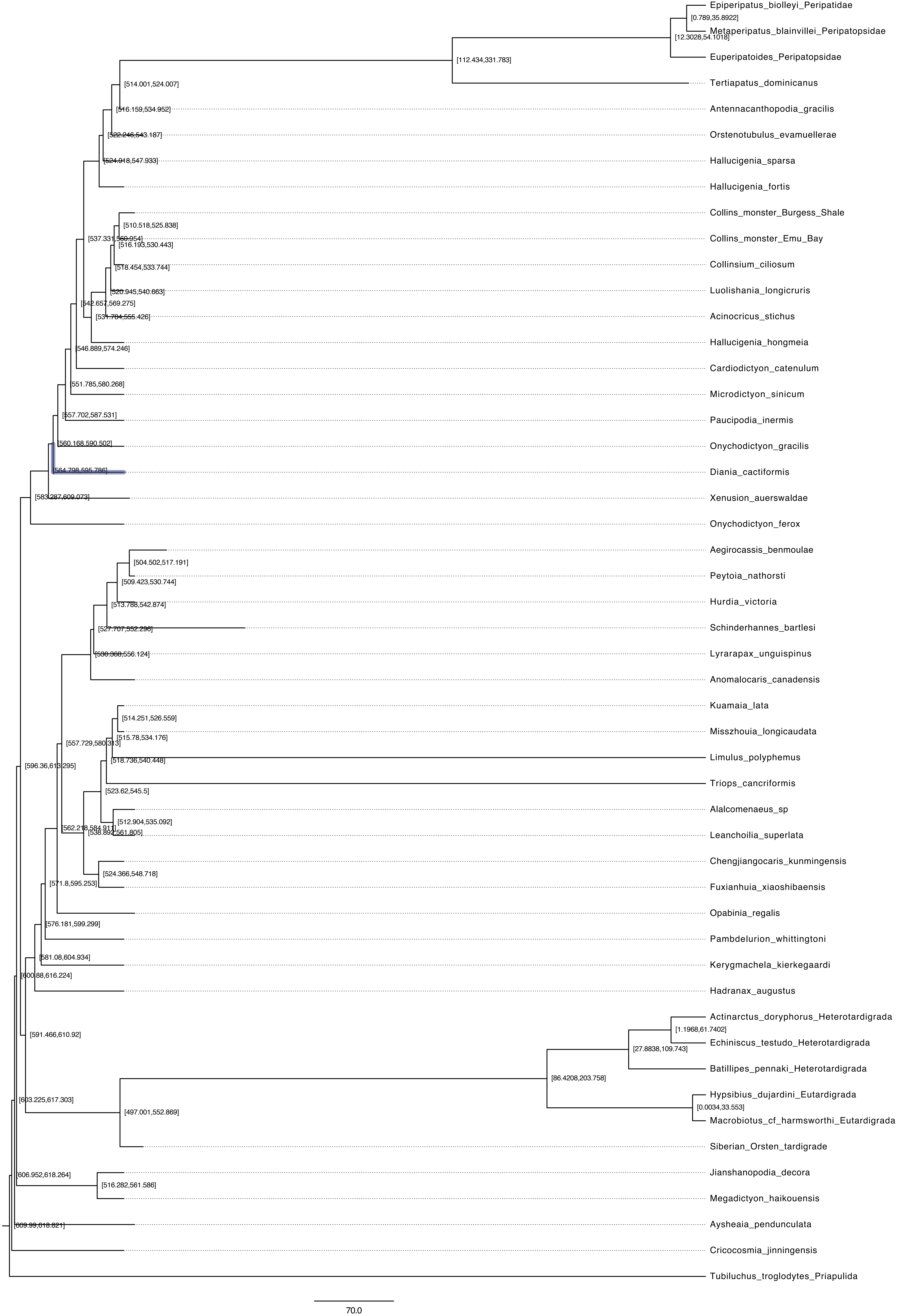

Epiperipatus_biolleyi_Peripatidae
[0.789,35.8922]
Metaperipatus_blainvillei_Peripatopsidae
[12.3028,54.1018]
Euperipatoides_Peripatopsidae
[112.434,331.783]
Tertiapatus_dominicanus
[514.001,524.007]
[516.159,534.952]
Antennacanthopodia_gracilis
[522.246,543.187]
Orstenotubulus_evamuellerae
[524.918,547.933]
Hallucigenia_sparsa
Hallucigenia_fortis
Collins_monster_Burgess_Shale
[510.518,525.838]
Collins_monster_Emu_Bay
[537.331,560.954]
[516.193,530.443]
Collinsium_ciliosum
[518.454,533.744]
Luolishania_longicruris
[520.945,540.663]
[542.657,569.275]
Acinocricus_stichus
[531.784,555.426]
Hallucigenia_hongmeia
[546.889,574.246]
Cardiodictyon_catenulum
551.785,580.268]
Microdictyon_sinicum
[557.702,587.531]
Paucipodia_inermis
[560.168,590.502]
Onychodictyon_gracilis
[564.798,595.786]
Diania_cactiformis
[583.287,609.073]
Xenusion_auerswaldae
Onychodictyon_ferox
Aegirocassis_benmoulae
[504.502,517.191]
Peytoia_nathorsti
[509.423,530.744]
Hurdia_victoria
[513.788,542.874]
[527.707,552.296]
Schinderhannes_bartlesi
[530.368,556.124]
Lyrarapax_unguispinus
Anomalocaris_canadensis
Kuamaia_lata
[514.251,526.559]
Misszhouia_longicaudata
[515.78,534.176]
[557.729,580.313]
Limulus_polyphemus
[518.736,540.448]
[596.36,613.295]
Triops_cancriformis
[523.62,545.5]
Alalcomenaeus_sp
[512.904,535.092]
[562.218,584.911]
[538.892,561.805]
Leanchoilia_superlata
Chengjiangocaris_kunmingensis
[524.366,548.718]
[571.8,595.253]
Fuxianhuia_xiaoshibaensis
Opabinia_regalis
[576.181,599.299]
Pambdelurion_whittingtoni
[581.08,604.934]
Kerygmachela_kierkegaardi
[600.88,616.224]
Hadranax_augustus
Actinarctus_doryphorus_Heterotardigrada
[1.1968,61.7402]
[591.466,610.92]
Echiniscus_testudo_Heterotardigrada
[27.8838,109.743]
Batillipes_pennaki_Heterotardigrada
[86.4208,203.758]
Hypsibius_dujardini_Eutardigrada
[603.225,617.303]
[497.001,552.869]
[0.0034,33.553]
Macrobiotus_cf_harmsworthi_Eutardigrada
Siberian_Orsten_tardigrade
[606.952,618.264]
Jianshanopodia_decora
[516.282,561.586]
Megadictyon_haikouensis
[609.99,618.821]
Aysheaia_pendunculata
Cricocosmia_jinningensis
Tubiluchus_troglodytes_Priapulida
70.0

Supplemental Figure 7: A fossil birth death tree of the Ecdysozoa, including both extinct and extant forms. Values expressed at each node are age in Ma.

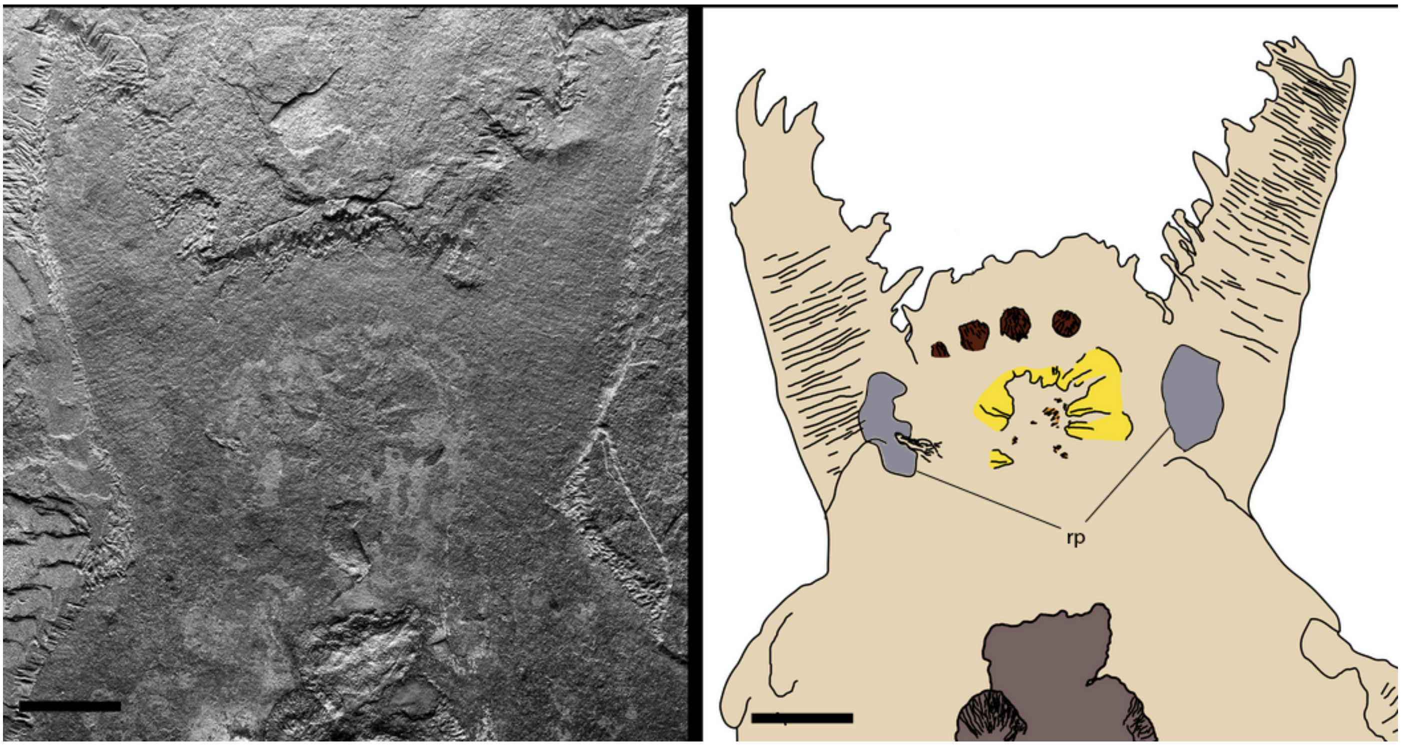
rp

Supplemental Figure 8: A specimen of *Pambdelurion whittingtoni* displaying distinct photoreflective patches, interpreted here as eye lobes. The right panel shows a diagrammatic represetation of the lobes on the specimen, depicted in the left panel.
